# Supplementary material for: LncRNA VEAL2 regulates PRKCB2 to modulate endothelial permeability in diabetic retinopathy
Source: EMBO J. 2021 Jun 28;40(15):e107134. doi: 10.15252/embj.2020107134 (PMC8327952; doi:10.15252/embj.2020107134)
Supplement: Supplementary file 2 — Appendix [file EMBJ-40-e107134-s009.pdf]

LncRNA VEAL2 regulates PRKCB2 to modulate endothelial permeability in diabetic retinopathy

Sehgal *et al*.

Short title: Regulation of Endothelial cells by *VEAL2*

Address for Correspondence:

Sridhar Sivasubbu, PhD., CSIR-Institute of Genomics and Integrative Biology, Delhi, 110025, India. E-Mail: s.sivasubbu@igib.res.in, sridhar@igib.in

Vinod Scaria, MBBS, PhD., CSIR-Institute of Genomics and Integrative Biology, Delhi, 110025, India. E-Mail: vinods@igib.in

Table of content:

1. **Page 2-7:** Supplementary results
2. **Page 8-40:** Appendix figures with legends S1-23
3. **Page 41-49:** Appendix tables S1,S2,S3,S4,S5,S6
4. **Page 50:** References

Supplementary Results

Discovery of Endothelial LncRNome

The purity of FACS was confirmed by morphological analysis and qRT-PCR based assay. Enrichment of EC markers: *fli1a* and *kdrl* was detected in EC population compared to NEC population, which indicated the efficiency of FACS (Appendix figure S1G). RNA was isolated from both the populations of cells and poly-A RNA capture and sequencing was performed to identify EC specific lncRNome (Figure 1A and Appendix figure S2). A total of ~22 million and ~25 million paired-end raw reads, each of 76 bp were generated from EC and NEC using Illumina GAIIx platform; over 80% of these reads mapped to the Zebrafish reference genome (Zv9/danRer7) (Appendix table S1). Overall 33,018 and 42,019 transcripts from EC and NEC, respectively, were assembled in a *de novo* manner.

The transcripts were analyzed through a custom-built bioinformatic pipeline to derive putative lncRNA (Figure 1A and Appendix figure S2). The reference sequences for the transcripts were retrieved from the UCSC Table Browser and the ones shorter than 200 bases were excluded. The open reading frames (ORFs) in all 6 frames were predicted for each transcript. Transcripts predicted to possess ORFs longer than 100 amino acids which could represent potential peptides were eliminated, yielding 9,524 transcripts. In order to compute the coding potential of the transcripts, the coding potential calculator (CPC) tool was used. After assessing the coding potential of all the transcripts, 6,502 potentially non-coding transcripts were retained with CPC score less than -1. Further, 384 transcripts with protein-coding isoforms were removed by intersection with the zebrafish RefSeq gene catalog. A total of 559 transcripts which showed significant hits in the Pfam protein database were eliminated in order to exclude transcripts with probable domains from known proteins. Additionally, BLASTx was performed on the repeat-masked transcript sequences against the zebrafish RefSeq protein database and 549 significant hits were omitted. In an attempt to place a secondary stringent filter for coding potential, PhyloCSF was used. A mere 6 transcripts were removed in this step, suggestive of an already well-culled

non-coding transcript dataset. Finally, the isoforms of all transcripts eliminated at each step of the pipeline were also excluded, to arrive at a final set of 4,897 putative lncRNA. A systematic classification of the predicted lncRNA was achieved by overlapping the putative transcripts with well-annotated RefSeq genes, identifying 925 promoter-associated lncRNA and 3,972 long intergenic RNA (lincRNA). The nearest protein-coding gene for each lncRNA has been listed (Dataset EV2).

In an attempt to provide an independent assessment of the protein-coding capacity of our lncRNA catalog, the translation efficiency scores were calculated on the basis of ribosomal occupancy on the predicted transcripts. For measuring the translational efficiency, the data from the previously reported ribosome profiling study performed across 8 stages of zebrafish development was retrieved. Translation Efficiency Scores (TES) were assigned to 26,829 zebrafish RefSeq genes (coding as well as non coding) along with the 3,866 novel predicted lncRNAs based on their estimated ribosomal occupancy. Our analysis showed that 2247 lncRNAs had a TES equal to zero. Large majority (91%) of the novel lncRNAs showed nil or negligible evidence of translation (TES < 0.001) compared to 82% of known noncoding RNAs and 1.4% of known protein-coding transcripts. The median TES for the novel lncRNAs (0) was found to be significantly lower than that of the protein-coding RefSeq genes (median TES:0.04), and comparable, if not lesser than that of the non coding RefSeq genes (median TES:0.001) (Figure 1B).

QC of endothelial versus non-endothelial datasets

The differential expression of some of the well studied vascular markers in EC and NEC samples were checked, including 6 upregulated genes (*fli1a, flt4, tbx6, kdrl, dlx2a* and *kdr/flk1*) and 2 down-regulated ones (*ptprn2, dlb*) all of which showed significant FPKM fold-change (Appendix table S6). Apart from these, a few more endothelial modulators like nephrosin (npsn) and hematopoietically expressed homeobox (hhex) were identified, which showed greater than 30-fold, and endothelium-specific receptor tyrosine kinase 1 (tie-1) and vascular associated protein

(vap) showed more than 4- fold elevated expression in EC. MicroRNA mir130c-1 which is known to be associated with heart development was also found to be EC-enriched (Appendix table S 10).

RACE of *veal2* and *VEAL2*

Total RNA from 24 hpf zebrafish was isolated using TRIzol (Invitrogen, USA) and 2ug of it was used for cDNA preparation of 5'& 3' RACE using manufacturer's protocol. *veal2*-GSP-1R was used to generate 5'RACE cDNA and adapter primer (dT) were used to generate 3'RACE cDNA. After tailing of 5'RACE cDNA using Tdt enzyme and dCTP, a gradient PCR with annealing temperature ranging from 60° C to 68° C was setup for 5' RACE using 5'RACE cDNA and *veal2*-GSP-2R. A very faint band was observed at 62° C which was gel extracted and purified. Two back to back nested PCR using *veal2*-GSP-3 & 4R were run at 62° C annealing temperature. The nested PCR of 5'RACE showed 4 different bands of size of approx 300bp, 350bp, 470bp and 580bp (Appendix figure S4C). All the bands were gel extracted individually and cloned in pCR 2.1-TOPO vector (Invitrogen, USA) and sanger sequencing was performed on it. The sequence of all the products were aligned with RNAseq annotated *veal2*. Only band at 580bp showed overlap with previously annotated *veal2* from RNAseq at 5'end with no extra nucleotide extension (Appendix figure S4D). The rest of the products were observed to be nonspecific as they did not align to the zebrafish genome.

Similarly, 3'RACE PCR was performed for *veal2* and *VEAL2* using *veal2*-GSP-1F and *VEAL2*-GSP-1F primers on respective cDNA prepared by oligo dT at 56°C annealing temperature. The 3' RACE PCR was followed by nested PCR using *veal2*-GSP-2F and *VEAL2*-GSP-2F. We observed a single band for both on gel which was gel extracted and cloned in pCR 2.1-TOPO vector (Invitrogen, USA) and sanger sequencing was performed on it. The sequenced nucleotides

were aligned with RNAseq annotated *veal2* and we observed extra 112bp nucleotides at the 3'end of *veal2* (Appendix figure S4A-B).

Anti-sense Morpholino-mediated knockdown of *veal2* perturbs developing vasculature

The functional relevance of *veal2* was evaluated using a splice-site targeting anti-sense modified oligo-morpholino (MO) (Appendix table S2), injected into one-cell stage zebrafish embryos (Appendix figure S6A). The downregulation of *veal2* expression inflicted vessel patterning defects in zebrafish larvae Appendix figure S6B-G), showing underdeveloped inter-segmental vessels (ISVs) and dorsal longitudinal anastomotic vessels (DLAVs), in a significant proportion of the surviving animals (50%, p-value<0.5E-4) (Appendix figure S6H). The intron retainment and downregulation of *veal2* was confirmed using end-point PCR and qRT-PCR (Appendix figure S6P-Q). We treated *veal2* downregulated animals with hemoglobin-binding O-dianisidine stain, and observed cranial hemorrhage (Appendix figure S6I-N). Few animals also displayed hemorrhage in the trunk region and eye. This showed that the loss-of-function of *veal2* alters endothelial permeability and vascular integrity.

To confirm that the altered vascular development and integrity defects are pertinent to the downregulation of *veal2* RNA, we attempted to rescue the phenotype by complementing with mature wild-type *veal2* RNA. The delivery of a cocktail of *in-vitro* synthesized mature wild-type *veal2* and anti-sense MO into single-cell zebrafish embryos resulted in 25% of the animals displaying hemorrhage, compared to 50% phenotype in animals injected with morpholino alone. Thus, about 50% rescue of the vascular integrity defects was achieved by the introduction of spliced *veal2* RNA exogenously (p-value<0.5E-3) (Appendix figure S6O).

Ectopic delivery of *veal2* induces excessive sprouting of sub-intestinal vessels

To assess the angiogenic activity of the lncRNA, we delivered the *in vitro* transcribed full-length *veal2* into the perivitelline cavity of 2.5-day old zebrafish larvae (Appendix figure S7A). The treated larvae developed abnormal sprouting of sub-intestinal vessels (SIV), as protrusions from the characteristic basket-like structure. The vascular sprouts were visualized by the Alkaline phosphatase staining method (Appendix figure S7C-D). The number of animals with excessively proliferating vessels increased significantly in a dose-dependent manner. (Appendix figure S7B). This data implicates a critical role for *veal2* in regulating angiogenesis.

Establishment of *veal2* knockout line

TALEN pairs targeting 5' and 3' ends of the *veal2* locus were injected separately into single cell of double transgenic *gib004Tg(fli1a:EGFP;gata1a:DsRed)* embryos. The TALEN pair targeting 5' end of *veal2* displayed specific vascular defects whereas the TALEN pair targeting 3' end led to severe developmental defects and apoptosis in F_0 somatic stage (Appendix figure S9A-B).

After the injection of 5' *veal2* TALEN, surviving phenotypic animals displaying cranial hemorrhage indicating blood vessel integrity defects (Appendix figure S10) were raised to adulthood and the genotypes were screened. Out of 8 F_0 animals, 4 (3 males and one female) displayed editing events at the *veal2* locus (Appendix figure S11). Particularly, one pair of the founders were in-crossed which gave rise to ~50 percent progeny with cranial hemorrhage (Appendix figure S12A-D) and the genotype was confirmed through capillary sequencing (Appendix figure S12E). The phenotypic progeny were raised to adulthood and genotyped after fin clipping. Of the 26 animals, 20 displayed indels at the targeted loci (Appendix figure S13). Ten animals showed the same 8 bp deletion (*veal2*Δ8). Possible off-target editing events were checked in 6 of these animals as described in the methods section (Appendix figure S14).

RAP-MS identified *veal2* interacting protein partner

From RAP-MS of *veal2* in zebrafish, 288 common (Dataset EV3) proteins obtained from triplicate experiments were analyzed using PANTHER Gene Ontology (GO) analysis tool (Muruganujan et al, 2019) (Appendix figure S16). About 10 proteins were predicted to be a part of the "Angiogenesis" pathway (Appendix table S5). Delta D (DLD), Fms-related Tyrosine Kinase 4 (FLT4), Eph receptor B3 (EPHB3) and Protein Kinase C beta b (Prkcbb) proteins were selected for validation, based on the availability of zebrafish antibodies for immunoblotting (Figure 3B).

Full length *veal2* is necessary to inhibit kinase activity of PRKCB

A small-molecule inhibitor of PRKCB2, Enzastaurin, was included as a positive control in the PRKCB kinase activity assay(Appendix figure S18A). To identify the functional domain of *veal2*, we synthesized 3 different smaller fragments of *veal2*-fragment-1 (1-146bp), *veal2*-fragment-2 (1-443bp), *veal2*-fragment-3 (1-754bp) and checked their individual effect on the activity of the human PRKCB2. None of the smaller fragments showed inhibition on the kinase activity of PRKCB2 (Appendix figure S18B), suggesting that the full-length *veal2* transcript is necessary for interaction with human PRKCB2 protein, at least *in-vitro*.

Appendix Figures and Legends

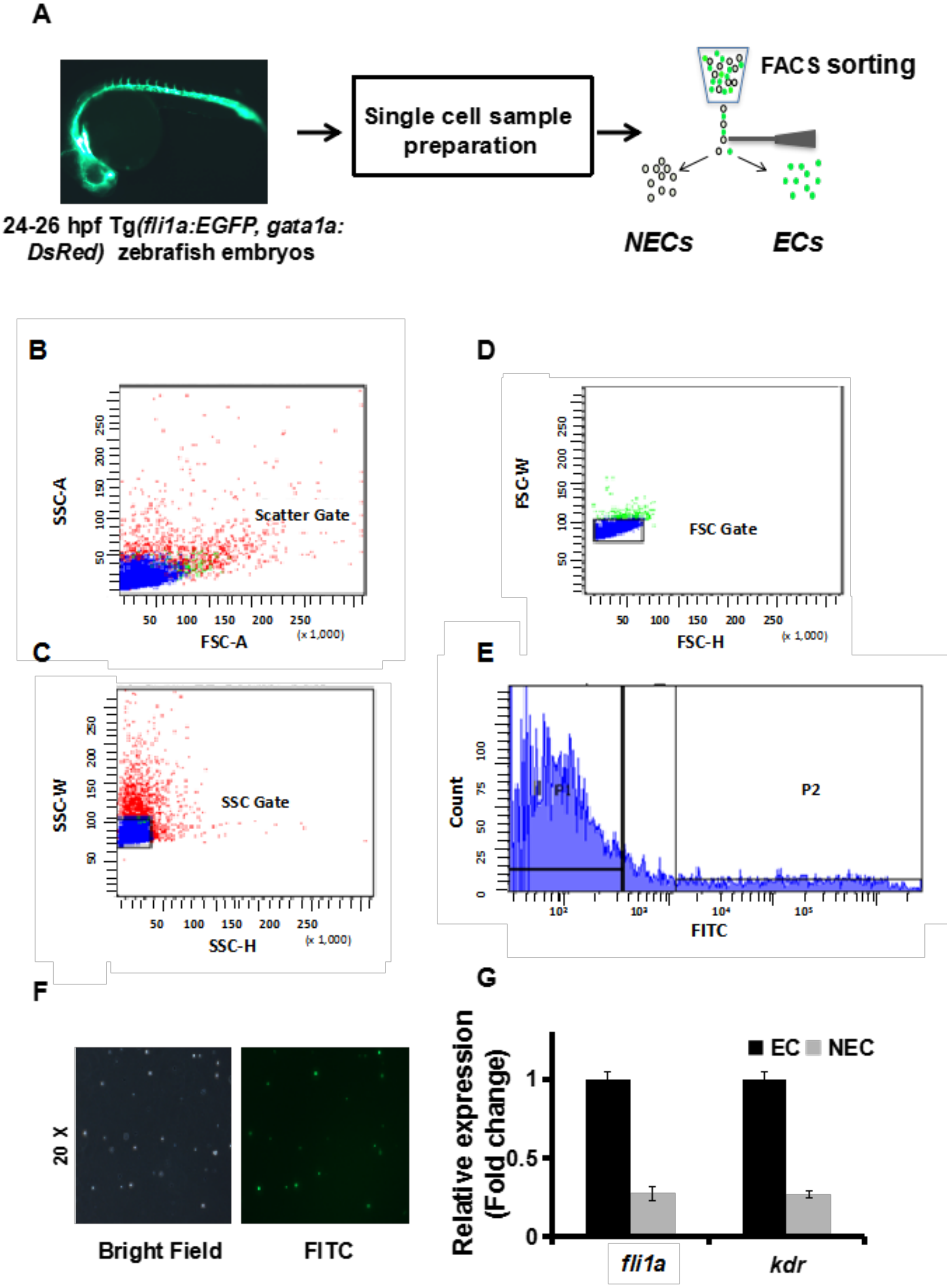

Appendix figure S1: Enrichment of GFP+ve endothelial cells from *gib004Tg(fli1a:EGFP;gata1a:DsRed)* zebrafish embryos

A-E. Fluorescence-activated cell sorting of heterogeneous cell populations prepared from dissociated 24-26 hpf double transgenic *gib004Tg(fli1a:EGFP;gata1a:DsRed)* zebrafish embryos. **A**. Live cells were selected by gating out the debris based on side (SSC-A) versus forward (FSC-A) scatter characteristics. **B-D**. Doublet cells excluded using height: area plot of side (B,C) and forward (D) scatter as they were out of the diagonal zone. **E**. Cells were segregated into two populations- cells displaying high FITC fluorescence signal (P2) and cell population with no FITC signal (P1).

F. Image acquisition of sorted cells under bright and FITC field (20X).

G. Quantification of relative expression of major vascular markers (*fli1a, kdr*) in GFP+ sorted EC versus GFP- NEC using RT-PCR.

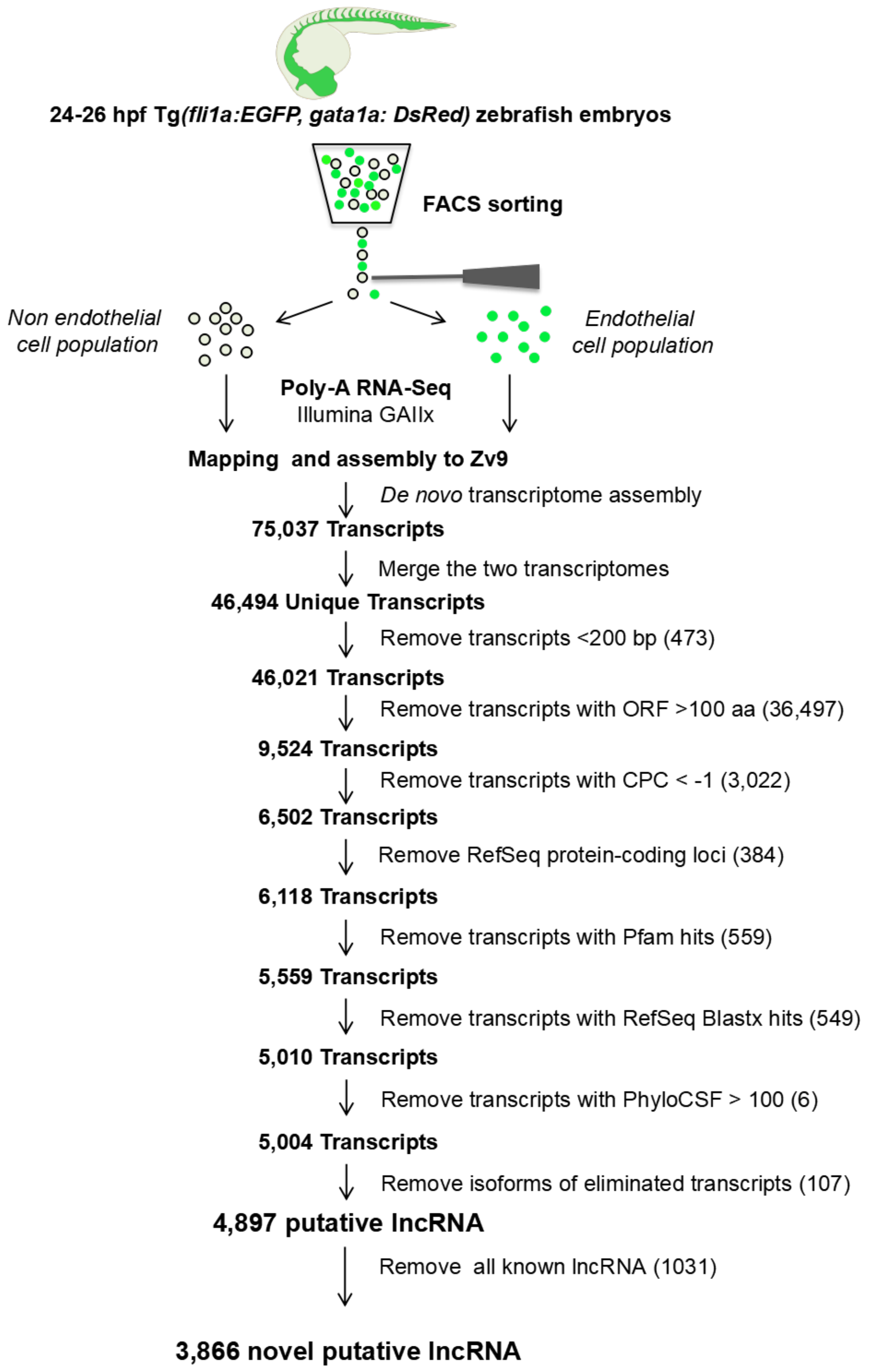

Appendix figure S2: Custom-built bioinformatic pipeline for the identification of lncRNome.

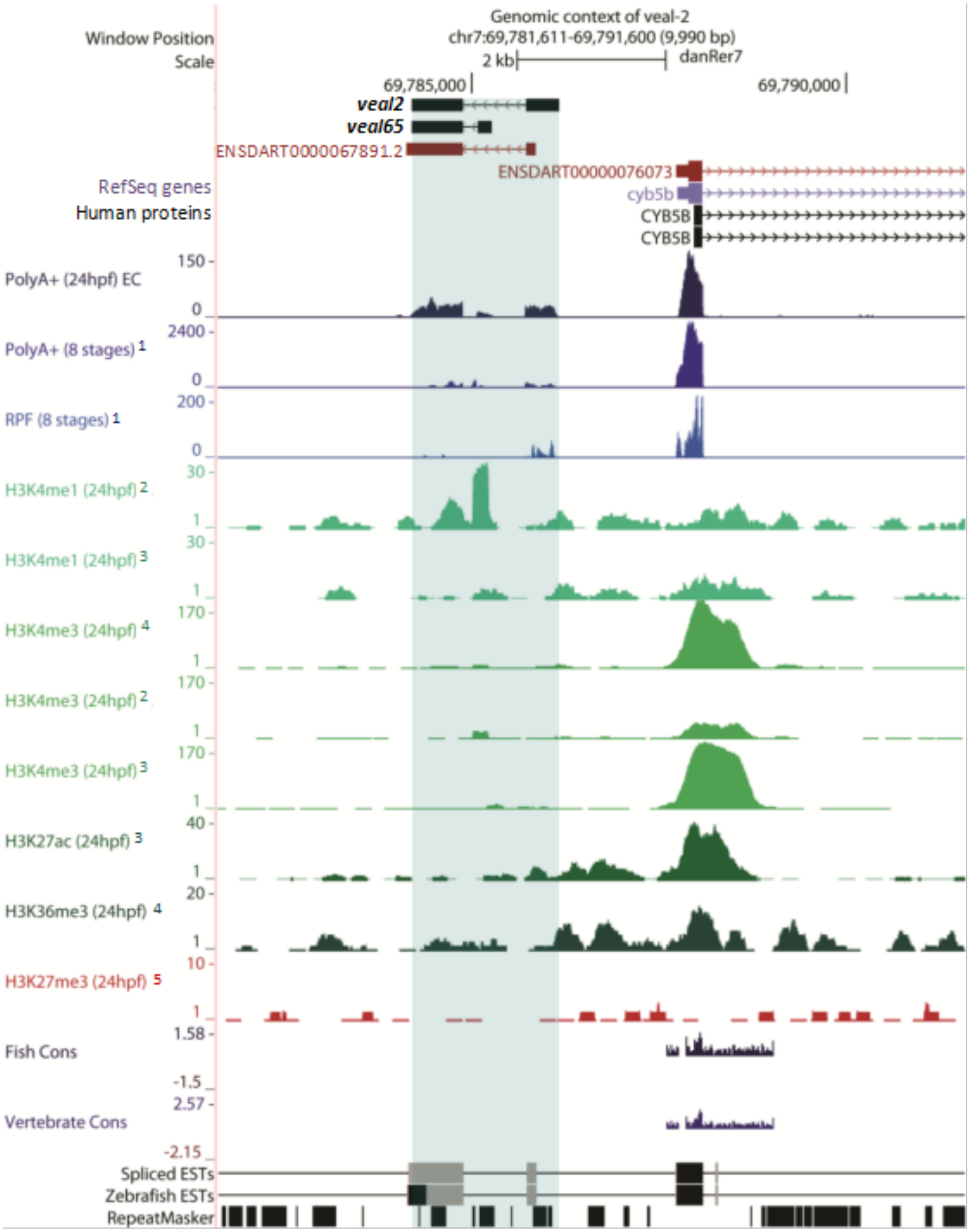
Genomic context of veal-2
Window Position
chr7:69,781,611-69,791,600 (9,990 bp)
Scale
2 kb
danRer7
69,785,000
69,790,000
veal2
veal65
ENSDART0000067891.2
ENSDART00000076073
RefSeq genes
cyb5b
Human proteins
CYB5B
CYB5B
PolyA+ (24hpf) EC
PolyA+ (8 stages) 1
RPF (8 stages) 1
H3K4me1 (24hpf) 2
H3K4me1 (24hpf) 3
H3K4me3 (24hpf) 4
H3K4me3 (24hpf) 2
H3K4me3 (24hpf) 3
H3K27ac (24hpf) 3
H3K36me3 (24hpf) 4
H3K27me3 (24hpf) 5
Fish Cons
Vertebrate Cons
Spliced ESTs
Zebrafish ESTs
RepeatMasker

Appendix figure S3: Genomic context of *veal2*

Genomic context of *veal2* locus along with endothelial RNA-Seq coverage, multi-stage ribo-seq and RNA-Seq coverage[15] and H3K4me1, H3K4me3, H3K27ac, H3K36me3, H3K27me3 chromatin marks (Aday et al, 2011, Bogdanovic et al, 2012, Irimia et al, 2012, Collins et al, 2012).

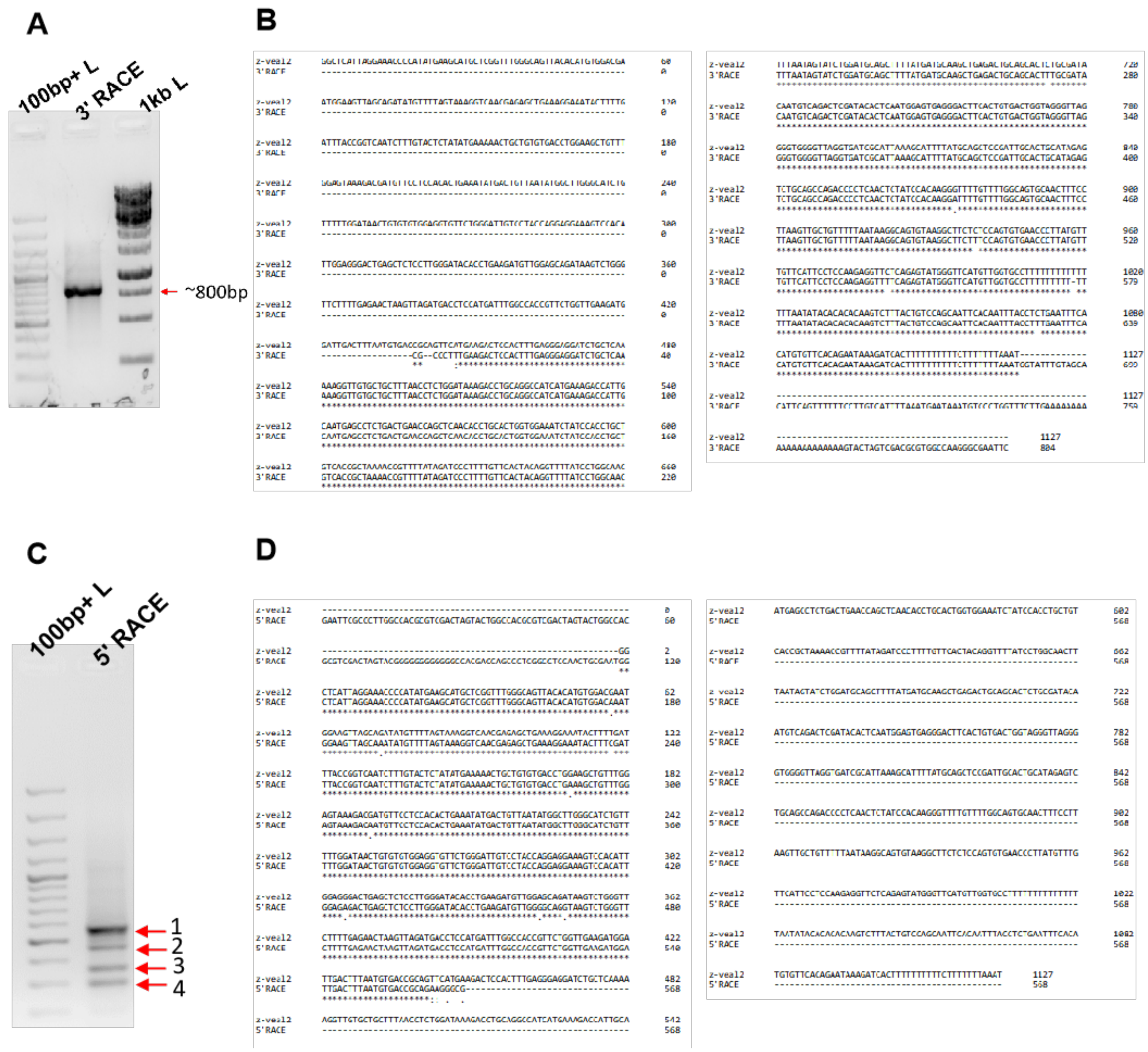

Appendix figure S4: Rapid amplification of cDNA ends identified full length sequence of *veal2*

A. Gel image representing the amplification products observed after nested PCR in 3'RACE.

B. Clustal omega based alignment of sequence identified by 3'RACE and *veal2* sequence.

C. Gel image representing the amplification products observed after nested PCR in 5'RACE.

D. Clustal omega based alignment of sequence identified by 5'RACE and *veal2* sequence.

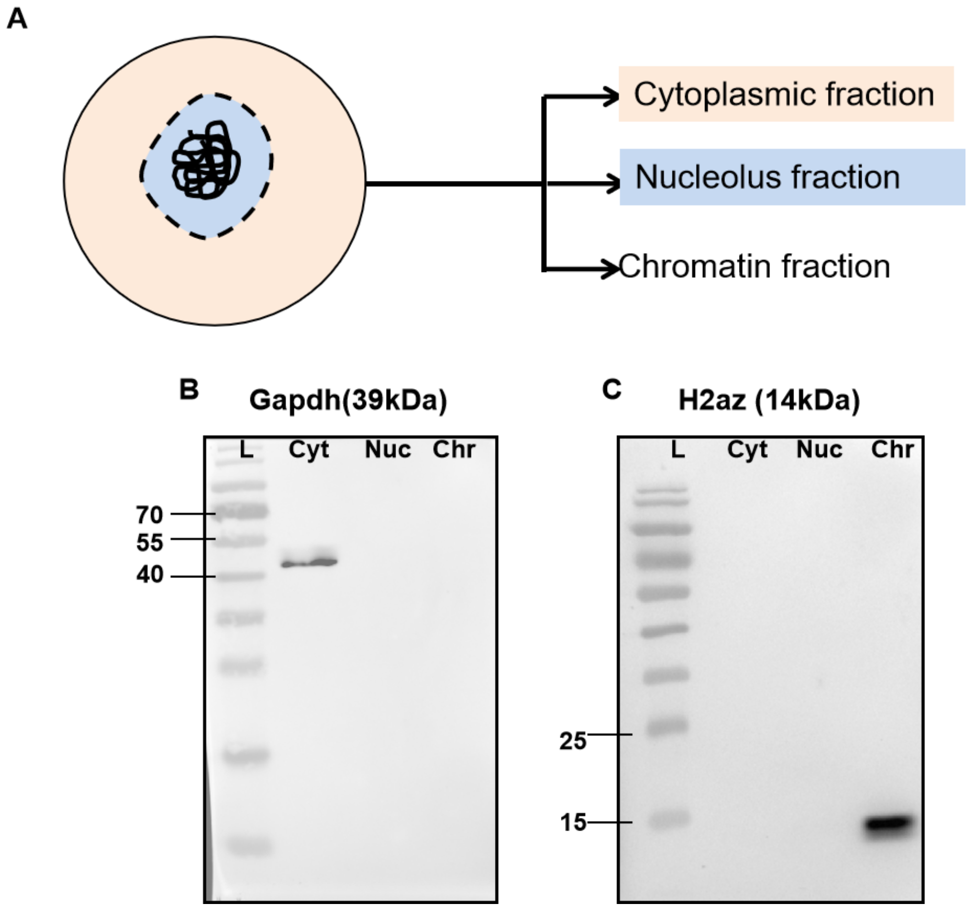

Appendix figure S5: Quality check of sub-cellular fractionation of cells of 1 dpf zebrafish embryos

A. Schematic representation of the different fractions of the cells collected to check the localization of *veal2*.

B. Representative image of the blot indicating presence of band only in the cytoplasmic fraction after probing with Gapdh antibody.

C. Representative image of the blot indicating presence of the band only in the chromatin fraction after probing with H2az antibody.

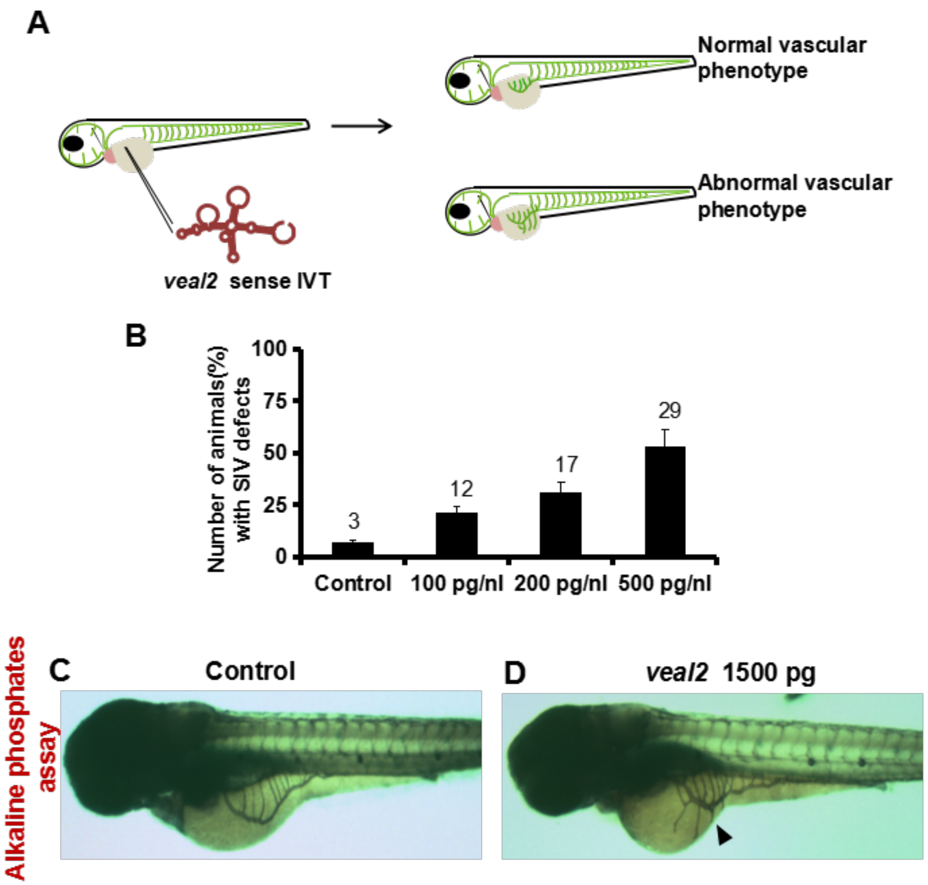

Appendix figure S6: Excessive SIV sprouting on over-expression of *veal2* in zebrafish hinted at a putative angiogenic role.

A. Schematic representation of microinjection of *in vitro* transcript of *veal2* in the perivitelline cavity of 2 dpf double transgenic *gib004Tg(fli1a:EGFP;gata1a:DsRed)* zebrafish embryo.

B. Bar graph represents the mean percentage of the number of embryos that displayed the phenotypic change after one-day post-injection of *veal2* IVT in the perivitelline cavity. Data collected from 3 different experiments and represented as mean percentage ± standard deviation.

C-D. Representation of the phenotype of embryos injected with *veal2* IVT compared to the embryos injected with vehicle control. Arrowhead represents the excessive sprout formation of sub intestinal vessels at the site of injection.

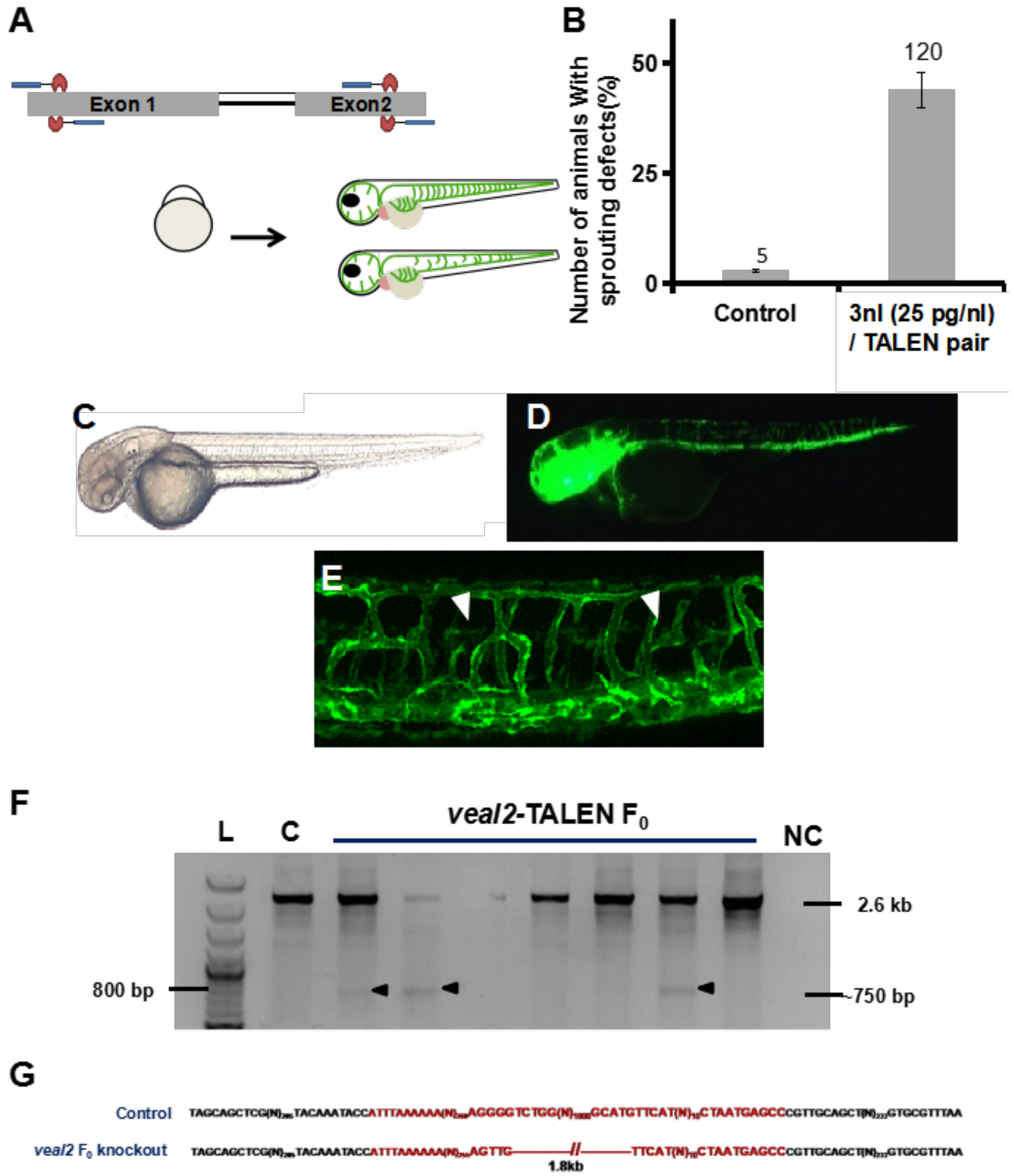

Appendix figure S7: Dual TALEN pair- mediated targeting of *veal2* locus caused deformities in vasculature development

A. Schematic representation of the TALEN design at both 5' and 3' of *veal2* and their co-injection into one cell of double transgenic *gib004Tg(fli1a:EGFP;gata1a:DsRed)* zebrafish embryos through microinjection for deleting the whole lncRNA locus. Injected animals were further screened at 2 dpf for any phenotypic changes.

B. Bar graph represents the number of animals in percentage which displayed the vascular sprouting defects in double transgenic *gib004Tg(fli1a:EGFP;gata1a:DsRed)* zebrafish embryos injected with dual pair of TALEN. Data collected from 3 different biological experiments were plotted as mean percentage ± standard deviation. Data labels represent the number of animals that showed the phenotype.

C-E. Representative images of the double transgenic *gib004Tg(fli1a:EGFP;gata1a:DsRed)* zebrafish embryos injected with dual pair of TALEN targeting *veal2* locus. **C-E** displaying vascular sprouting defects at 2 dpf under bright field and EGFP filters. **C-D-** 5X magnification. **E-** 20X magnification. Arrowheads represent the presence of underdeveloped intersegmental vessels due to vascular sprouting defects.

F. Gel showing different amplicons of *veal2* locus amplified from 1 control embryo and 7 tested embryos injected with dual pair of TALENs. Arrowheads represent an additional band in 3 out of 7 tested embryos due to the large deletion.

G. Sequence information of *veal2* locus of control embryo and embryo injected with dual pair of TALENs identified using capillary sequencing. A deletion of 1.8kb within the *veal2* locus was confirmed in the embryo injected with dual pair of TALENs.

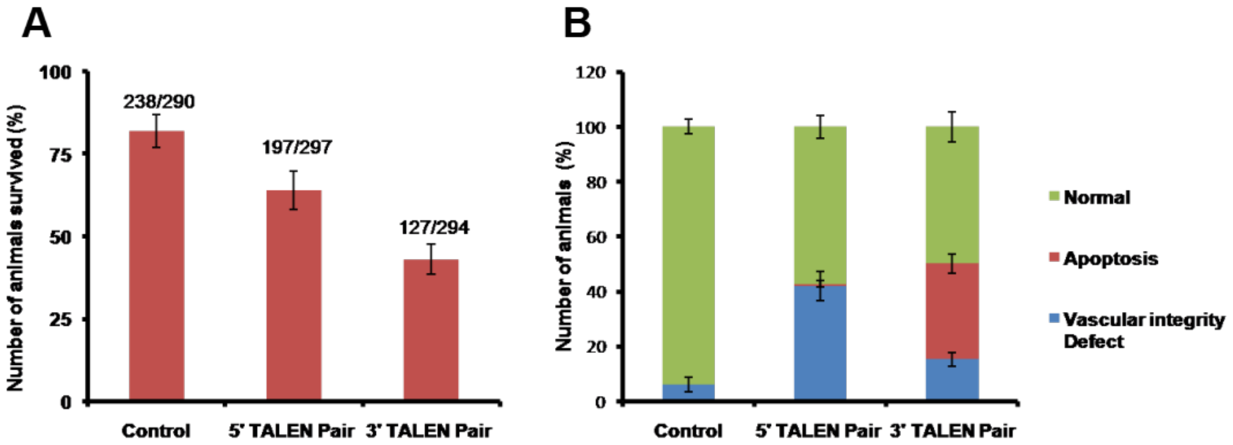

Appendix figure S8: TALEN targeting the 5' of *veal2* is more specific to cause vascular defects compared to the TALEN targeting 3' end

A. Bar graph representing the survival of the embryos injected with vehicle control (nuclease-free water), TALEN pair targeting 5' end and 3' end. Data was collected from 3 independent experiments and represented as mean percentage ± standard deviation. Data labels represent the number of the animals which survived out of the total number of animals across the three experiments.

B. Bar graph representing the number of the embryos which displayed the phenotypes when injected with vehicle control (nuclease-free water), TALEN pair targeting 5' end and 3' end. Data was collected from 3 independent biological experiments and represented as mean percentage ± standard deviation.

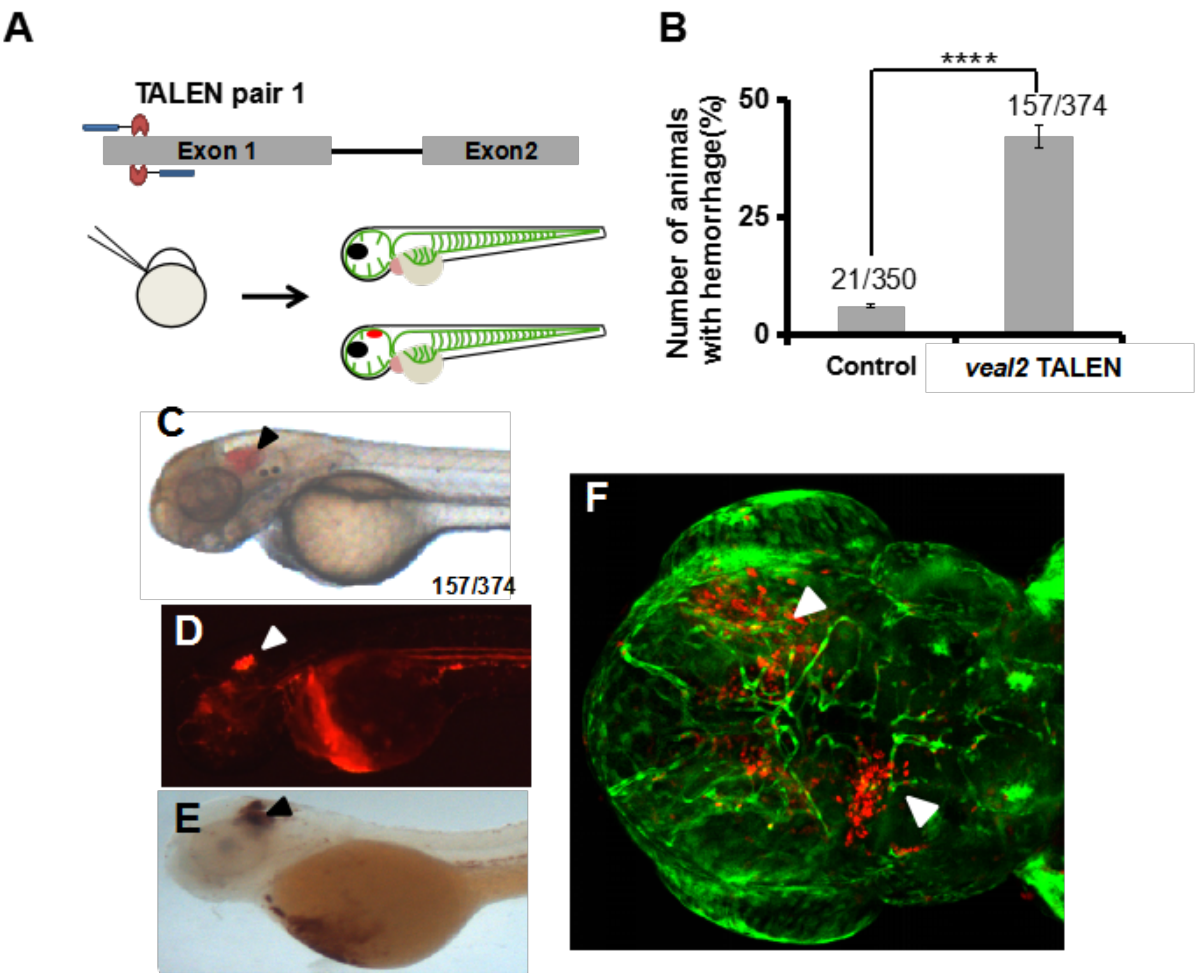

Appendix figure S9: TALEN based genome- editing of *veal2* locus caused vascular integrity defects and hemorrhage in zebrafish embryos.

A. Schematic representation of the design and target sites of TALEN arms on 5' end of *veal2* locus and its injection into one cell staged of double transgenic *gib004Tg(fli1a:EGFP;gata1a:DsRed)* zebrafish embryos through microinjection. The injected embryos were further screened at 2 dpf for any phenotypic changes.

B. Bar graph representing the number of animals displaying hemorrhage in F_0 animals. Data was obtained from more than 3 experiments and represented as mean percentage ± standard deviation.

C-F. Representative images of the double transgenic *gib004Tg(fli1a:EGFP;gata1a:DsRed)* zebrafish embryos injected with 3 nl of 25 pg/nl per TALEN arm targeting the *veal2* locus. Embryos are displaying hemorrhage due to vascular integrity defects at 2 dpf under bright field and EGFP filters. Arrowheads represent the hemorrhage due to vascular integrity defects. **C-E-** 5X magnification and F**-** 20X magnification.

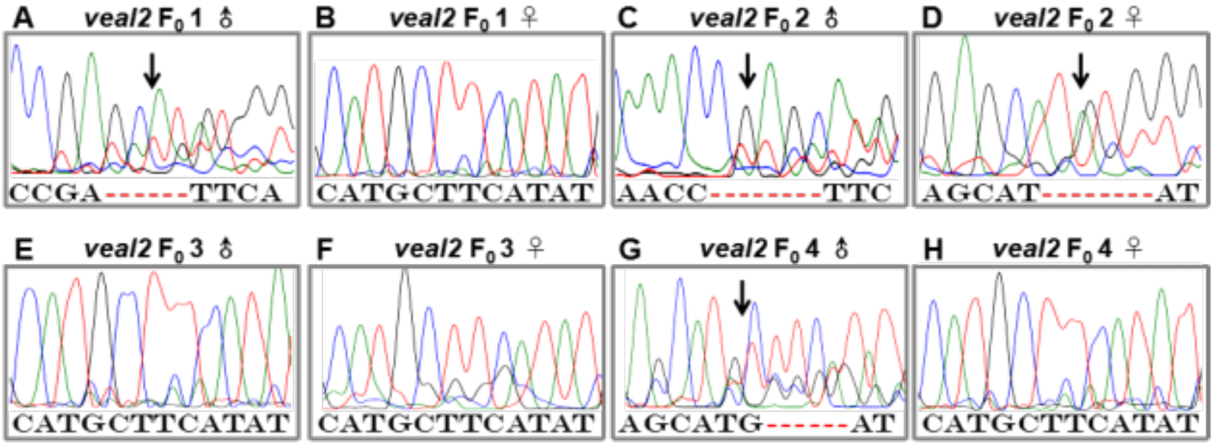

Appendix figure S10: Capillary sequencing confirmed the presence of indels in *veal2* locus in fin-clip DNA from F_0 founders.

A-H. Chromatogram snapshots of the spacer region of the targeted region of 5' end of *veal2* along with the ID given to the animals and the sequence information. Arrows indicate the sites from

where the mutations start.

A

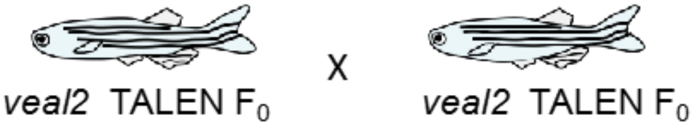

Number of embryos	Phenotypic embryos	Genotype confirmed	No of animals grown	Animals survived after 3 months
196	94(48%)	8 out of 8	90	48

B C D

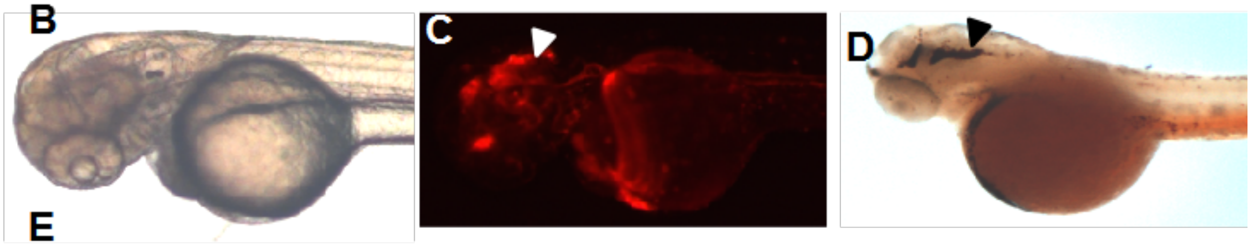

E

S. No.	Sequence	Indel	Multiple
WT	CCACATGTGTAACTGCCCAAA*CCGAGCATGCTTCATAT*GGGTTT		
Mut1	CCACATGTGTAACTGCCCAAACCGAGCATGCATGCTTCATATGGGTTT	+4	X 2
Mut2	CCACATGTGTAACTGCCCAAACCGAGCATGCTTGC-TATGGGTTT	+1,-1	
Mut3	CCACATGTGTAACTGCCCAAACCGAGCATGCATGCTTCATATGGGTTT	+4	X 4
Mut4	CCACATGTGTAACTGCCCAAACC--------TTCATATGGGTTT	-8	X 6
Mut5	CCACATGTGTAACTGCCCAAACC-------CTTCATATGGGTTT	-8	
Mut6	CCACATGTGTAACTGCCCAAACCGAGCATGCATGCTTCATATGGGTTT	+4	x 3

Appendix figure S11: Transmission of the genotype and phenotype to next-generation upon outcross of founder animals reveals the pertinent role of *veal2*.

A. Schematic representation of the cross of *veal2* F_0 founder zebrafish along with summary of the cross.

B-D. Representative images of the *veal2* F_1 animals which displayed the hemorrhage phenotype at 2 dpf as observed under bright field, mRFP filters and animals stained with O-dianisidine under the bright field. Arrowheads represent the hemorrhage due to the vascular integrity defects.

E. List of sequences showing various indels in the *veal2* locus transmitted to the next generation of F_1 *veal2* zebrafish embryos from the out-cross of the founder *veal2* mutant with double transgenic *gib004Tg(fli1a:EGFP;gata1a:DsRed)* control animals.

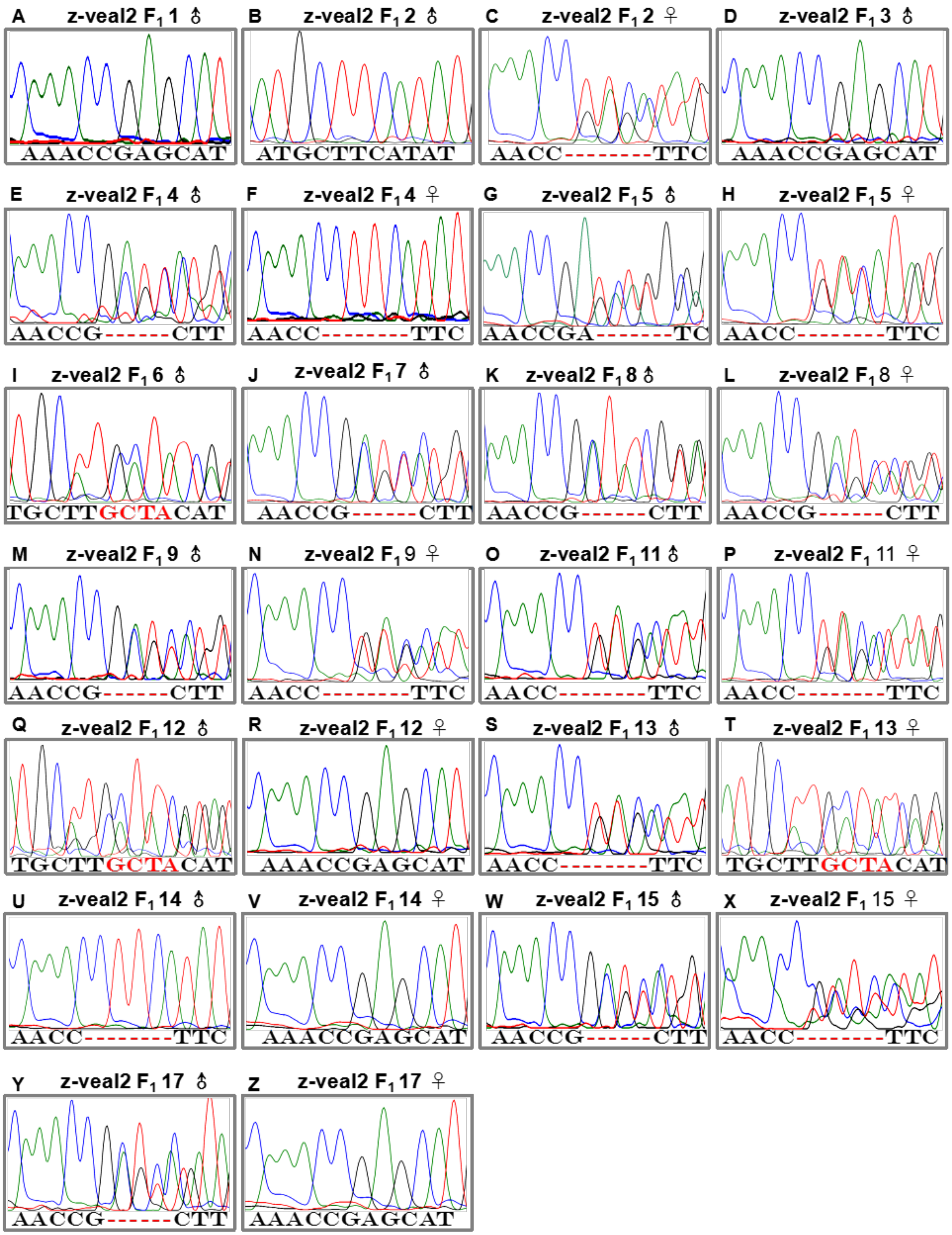
A z-veal2 F_1 1 ♂
AAACCGAGCAT
B z-veal2 F_1 2 ♂
ATGCTTCATAT
C z-veal2 F_1 2 ♀
AACC---------TTC
D z-veal2 F_1 3 ♂
AAACCGAGCAT
E z-veal2 F_1 4 ♂
AACCG------CTT
F z-veal2 F_1 4 ♀
AACC---------TTC
G z-veal2 F_1 5 ♂
AACCGA-------TC
H z-veal2 F_1 5 ♀
AACC---------TTC
I z-veal2 F_1 6 ♂
TGCTTGCTACAT
J z-veal2 F_1 7 ♂
AACCG------CTT
K z-veal2 F_1 8 ♂
AACCG------CTT
L z-veal2 F_1 8 ♀
AACCG------CTT
M z-veal2 F_1 9 ♂
AACCG------CTT
N z-veal2 F_1 9 ♀
AACC---------TTC
O z-veal2 F_1 11 ♂
AACC---------TTC
P z-veal2 F_1 11 ♀
AACC---------TTC
Q z-veal2 F_1 12 ♂
TGCTTGCTACAT
R z-veal2 F_1 12 ♀
AAACCGAGCAT
S z-veal2 F_1 13 ♂
AACC---------TTC
T z-veal2 F_1 13 ♀
TGCTTGCTACAT
U z-veal2 F_1 14 ♂
AACC---------TTC
V z-veal2 F_1 14 ♀
AAACCGAGCAT
W z-veal2 F_1 15 ♂
AACCG------CTT
X z-veal2 F_1 15 ♀
AACC---------TTC
Y z-veal2 F_1 17 ♂
AACCG------CTT
Z z-veal2 F_1 17 ♀
AAACCGAGCAT

Appendix figure S12: Capillary sequencing confirmed the presence of indels in *veal2* locus in fin-clipped F_1 founder zebrafish animals.

A-Z. Chromatogram snapshots of the spacer region of the targeted region of 5' end of *veal2* typed in 26 *veal2* edited F_1 animals and the sequence information. Arrows indicate the sites from where the mutations start.

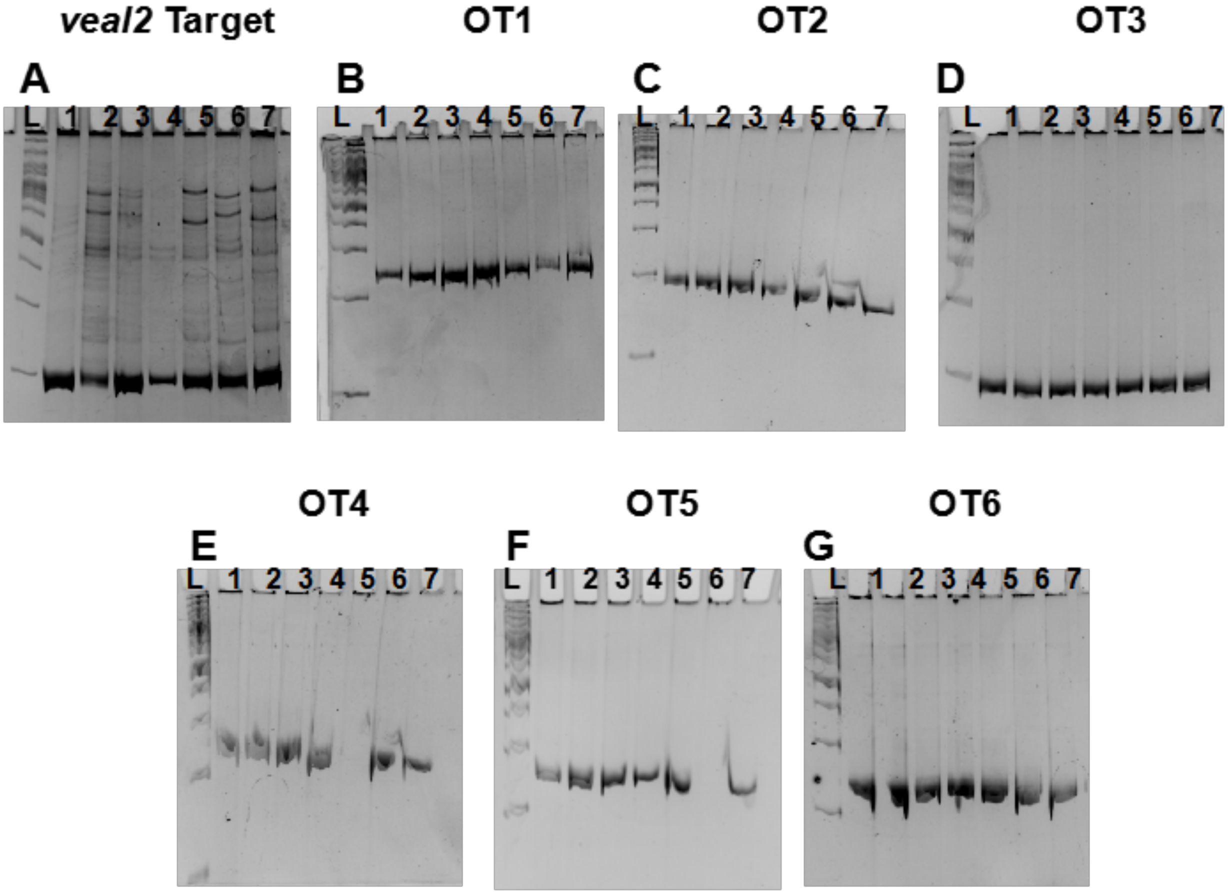

L = 100bp ladder, 1 = Control ♂, 2 = *veal2* F_1 11 ♂, 3 = *veal2* F_1 11 ♀
4 = *veal2* F_1 5 ♂, 5 = *veal2* F_1 5 ♀, 6 = *veal2* F_1 13 ♂, 7 = *veal2* F_1 2 ♀

Appendix figure S13: Heteroduplex mobility assay confirmed absence of off-target editing events at the queried loci in *veal2*$^{gib005\Delta8/+}$ zebrafish.

HMA of 7 amplified loci in control and 6 tested *veal2*$^{gib005\Delta8/+}$ animals on PAGE gel. Absence of heteroduplexes in all the queried loci in *veal2*$^{gib005\Delta8/+}$ animals indicate absence of off targets.

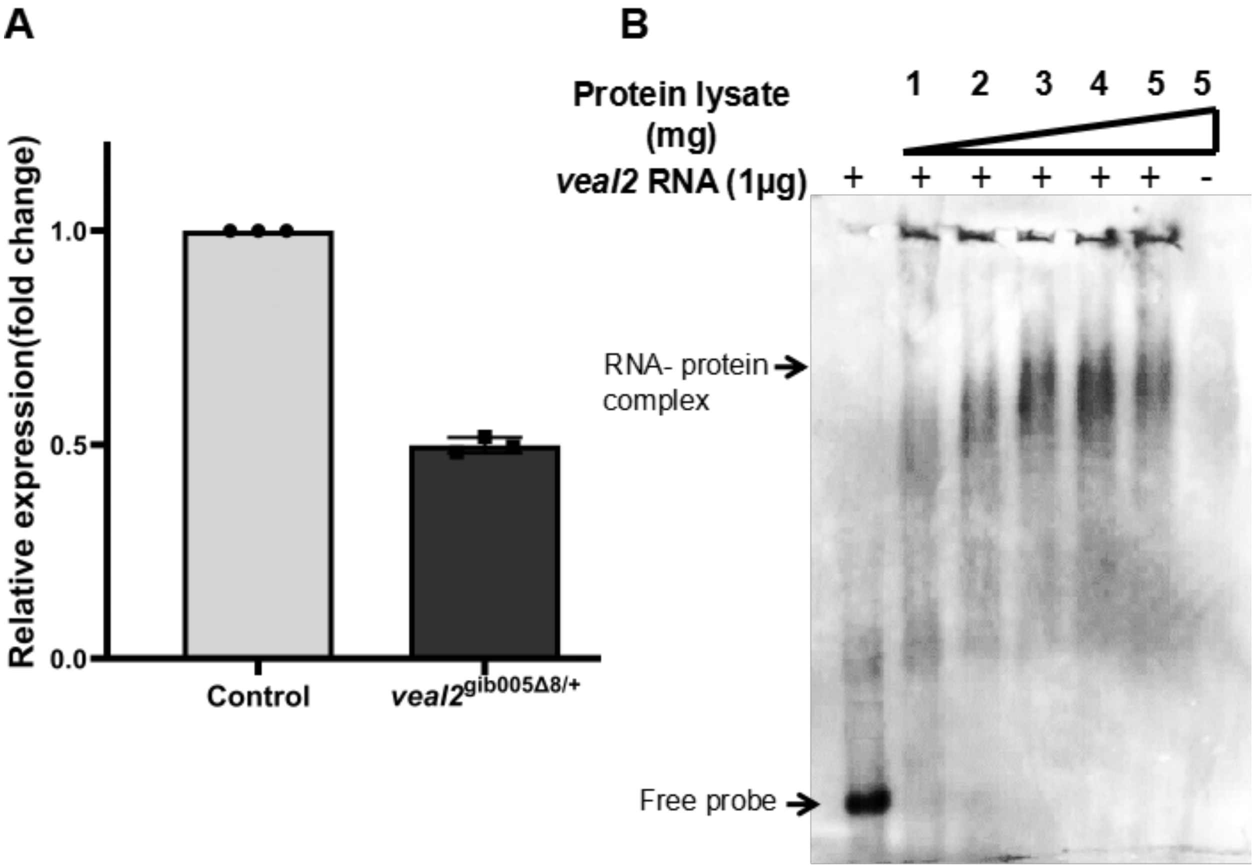

Appendix figure S14: Electrophoretic Mobility Shift Assay (EMSA) predicted the protein binding affinity of *veal2*.

A. Bar graph representing relative expression of wild type *veal2* allele in control and in *veal2*$^{gib005\Delta8/+}$ animals showing reduction in expression significantly. Data is acquired from 3 different biological replicates and shown as mean fold change values ± standard deviation.

B. The blot represents the mobility shift of *veal2* in the presence of different concentrations of the total protein isolated from 1 dpf zebrafish embryos. Arrows indicate the presence of *veal2* probed by an anti-sense DIG-labeled probe.

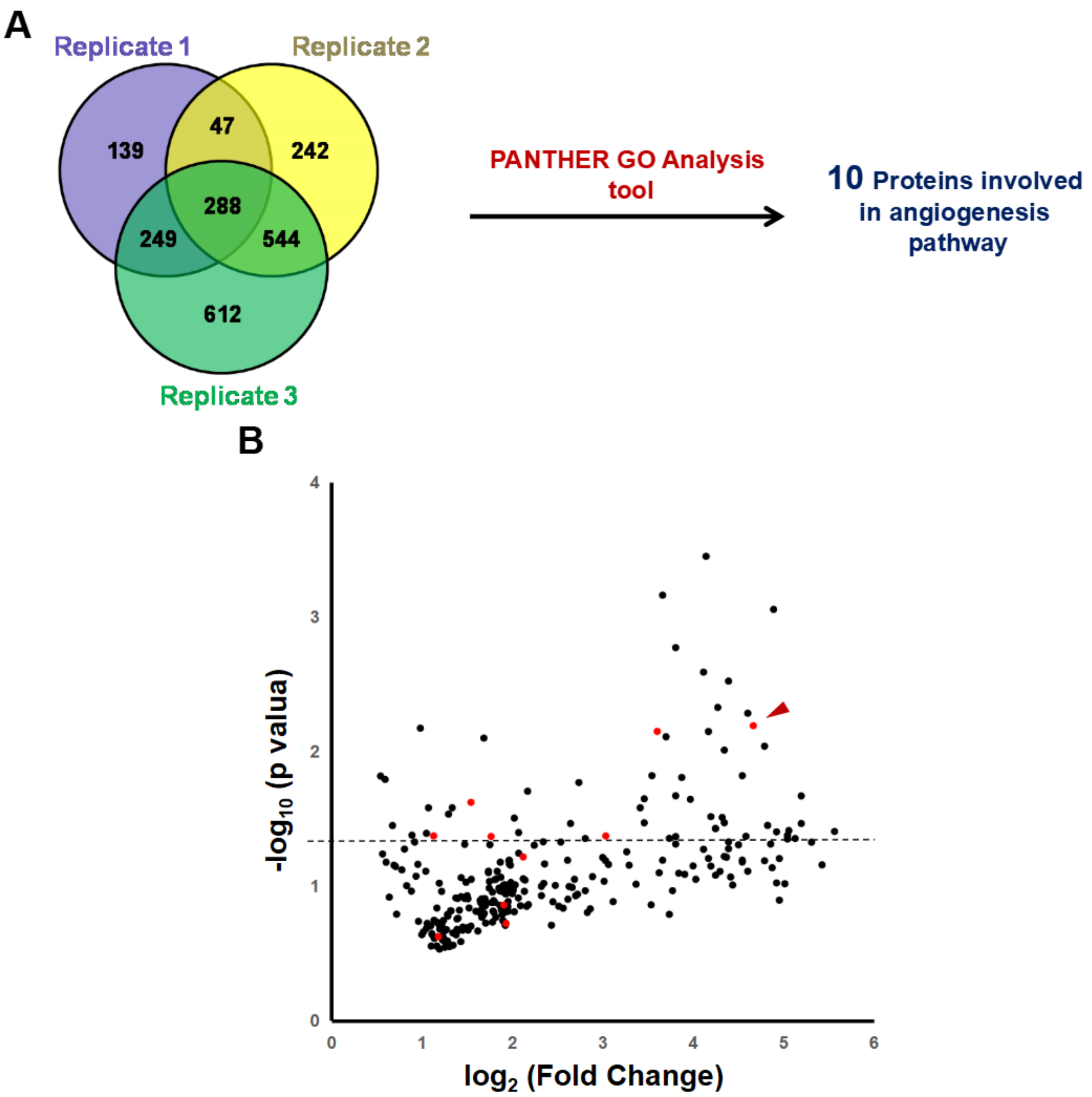

Appendix figure S15: A. The Venn diagram represents the overlap between the proteins identified as interacting with *veal2* from 3 different replicates. Using PANTHER GO analysis tools proteins enriched in "Angiogenesis" pathway were selected for validation.

B. Dot plot graph representing the fold change ($\log_2$) of the proteins across *veal2* pull-down and no probe control. Dots highlighted in red color are the proteins which are the part of "Angiogenesis" pathway. The arrowhead indicates the Prkcbb protein. The dotted line indicates the p value of 0.05.

A

```
sp|P05771|PRKCB_HUMAN        MADPAAGPPPSEGEE---STVRFARKGALRQKNVHEVKNHKFTARFFKQPTFCSHCTDFI   57
sp|Q7SY24|PRKCB_Zebrafish    MAE----PANSDGEERSAPMRGFARQGALRQKNVHEVKNHKFIARFFKQPTFCSHCTDFI   56
                             **:    *  *:***        ***:**************** *****************

sp|P05771|PRKCB_HUMAN        WGFGKQGFQCQVCCFVVHKRCHEFVTFSCPGADKGPASDDPRSKHKFKIHTYSSPTFCDH   117
sp|Q7SY24|PRKCB_Zebrafish    WGFGKQGFQCQVCCFVVHKRCHEFVTFSCPGADKGPASDDPRSKHKFKVHTYSSPTFCDH   116
                             ************************************************:***********

sp|P05771|PRKCB_HUMAN        CGSLLYGLIHQGMKCDTCMMNVHKRCVMNVPSLCGTDHTERRGRIYIQAHIDRDVLIVLV   177
sp|Q7SY24|PRKCB_Zebrafish    CGSLLYGLIHQGMRCDHCMMNIHKRCVANVPSLCGTDHTERRGRIQITAEIKNNVLTVSI   176
                             *************:** ****:***** ***************** * *.*..:** * :

sp|P05771|PRKCB_HUMAN        RDAKNLVPMDPNGLSDPYVKLKLIPDPKSESKQKTKTIKCSLNPEWNETFRFQLKESDKD   237
sp|Q7SY24|PRKCB_Zebrafish    KEAKNLVPMDPNGLSDPYVKLKLIPDPKSESKQKTKTIKCCLNPTWNETFTFNLKESDKD   236
                             ::**************************************.*** ***** *:*******

sp|P05771|PRKCB_HUMAN        RRLSVEIWDWDLTSRNDFMGSLSFGISELQKASVDGWFKLLSQEEGEYFNVPVPPEGSEA   297
sp|Q7SY24|PRKCB_Zebrafish    RRLSVEIWDWDLTSRNDFMGSLSFGISELQKQGVDGWFKLLSQEEGEYFNVPVPPEGEEG   296
                             ******************************* .************************ *.

sp|P05771|PRKCB_HUMAN        NEELRQKFERAKISQGTKVPEEKTTNTVSKFDNNGNRDRMKLTDFNFLMVLGKGSFGKVM   357
sp|Q7SY24|PRKCB_Zebrafish    NEELRQKFERAKIGPS--KTDGSSSNAISKFDSNGNRDRMKLSDFNFLMVLGKGSFGKVM   354
                             *************. .    :  .::*::****.*********:*****************

sp|P05771|PRKCB_HUMAN        LSERKGTDELYAVKILKKDVVIQDDDVECTMVEKRVLALPGKPPFLTQLHSCFQTMDRLY   417
sp|Q7SY24|PRKCB_Zebrafish    LAERKGADELFAIKILKKDVVIQDDDVECTMVEKRVLALSGKPPFLTQLHSCFQTMDRLY   414
                             *:****:***:*:************************** ********************

sp|P05771|PRKCB_HUMAN        FVMEYVNGGDLMYHIQQVGRFKEPHAVFYAAEIAIGLFFLQSKGIIYRDLKLDNVMLDSE   477
sp|Q7SY24|PRKCB_Zebrafish    FVMEYINGGDLMYHIQQVGKFKEPHAVFYAAEIAIGLFFLHSKGVIYRDLKLDNVMLDAE   474
                             *****:*************:********************:***:*************:*

sp|P05771|PRKCB_HUMAN        GHIKIADFGMCKENIWDGVTTKTFCGTPDYIAPEIIAYQPYGKSVDWWAFGVLLYEMLAG   537
sp|Q7SY24|PRKCB_Zebrafish    GHIKIADFGMCKENMLDGVTTKTFCGTPDYIAPEIIAYQPYGKSVDWWAFGVLLYEMLAG   534
                             **************: ********************************************

sp|P05771|PRKCB_HUMAN        QAPFEGEDEDELFQSIMEHNVAYPKSMSKEAVAICKGLMTKHPGKRLGCGPEGERDIKEH   597
sp|Q7SY24|PRKCB_Zebrafish    QPPFDGEDEDELFQSIMEHHVSYPKSMSKEAVAICKGLMTKHPGKRLGCGPEGERDIREH   594
                             * **:**************:*:***********************************:**

sp|P05771|PRKCB_HUMAN        AFFRYIDWEKLERKEIQPPYKPKARDKRDTSNFDKEFTRQPVELTPTDKLFIMNLDQNEF   657
sp|Q7SY24|PRKCB_Zebrafish    GFFRYMDWEKLEHREVQPPFKPKACG-RDAENFDRFFTRHPPVLTPPDQEVIMNLDQDEF   653
                             .****:******::*:***:**** . **:.***: ***:*  *** *: .******:**

sp|P05771|PRKCB_HUMAN        AGFSYTNPEFVINV---    671
sp|Q7SY24|PRKCB_Zebrafish    EGFSFINPEFPAMEAQS    670
                              ***: ****
```

B

```
sp|P05771|PRKCB_HUMAN          SKHKFKIHTYSSPTFCDHCGSLLYGLIHQGMKCDTCMMNVHKRCVMNVPS
tr|H2QAS7|PRKCB_Chimpanzee     SKHKFKIHTYSSPTFCDHCGSLLYGLIHQGMKCDTCMMNVHKRCVMNVPS
tr|H0V0W8|PRKCB_Guinea         SKHKFKIHTYSSPTFCDHCGSLLYGLIHQGMKCDTCMMNVHKRCVMNVPS
sp|P68403|PRKCB_RAT            SKHKFKIHTYSSPTFCDHCGSLLYGLIHQGMKCDTCMMNVHKRCVMNVPS
sp|P68404|PRKCB_MOUSE          SKHKFKIHTYSSPTFCDHCGSLLYGLIHQGMKCDTCMMNVHKRCVMNVPS
sp|Q7SY24|PRKCB_Zebrafish      SKHKFKVHTYSSPTFCDHCGSLLYGLIHQGMRCDHCMMNIHKRCVANVPS
                               ******:************************:** ****:***** ****
```

Appendix figure S16: Sequence conservation of PRKCB protein across vertebrates

A. Clustal omega alignment of Zebrafish Prkcbb and Human PRKCB2 protein. Color labels indicate conserved domains of PRKCB protein. Grey indicates C1-A, Yellow- C1-B, Dark green- C2, Cyan- protein kinase domain. Residues highlighted in green in C1-B domain are the amino acids which are known to interact directly with Diacylglycerol (DAG) (Leonard et al, 2011). Amino acids highlighted in red in C1-B domain are the residues which interact with *veal2* predicted by *in silico* docking.

B. Clustal omega alignment of C1B domain of PRKCB protein of Human, Chimpanzee, Guinea pig, Rat, Mouse and Zebrafish. DAG binding and *veal2* binding residues are shown in green and red colours, respectively.

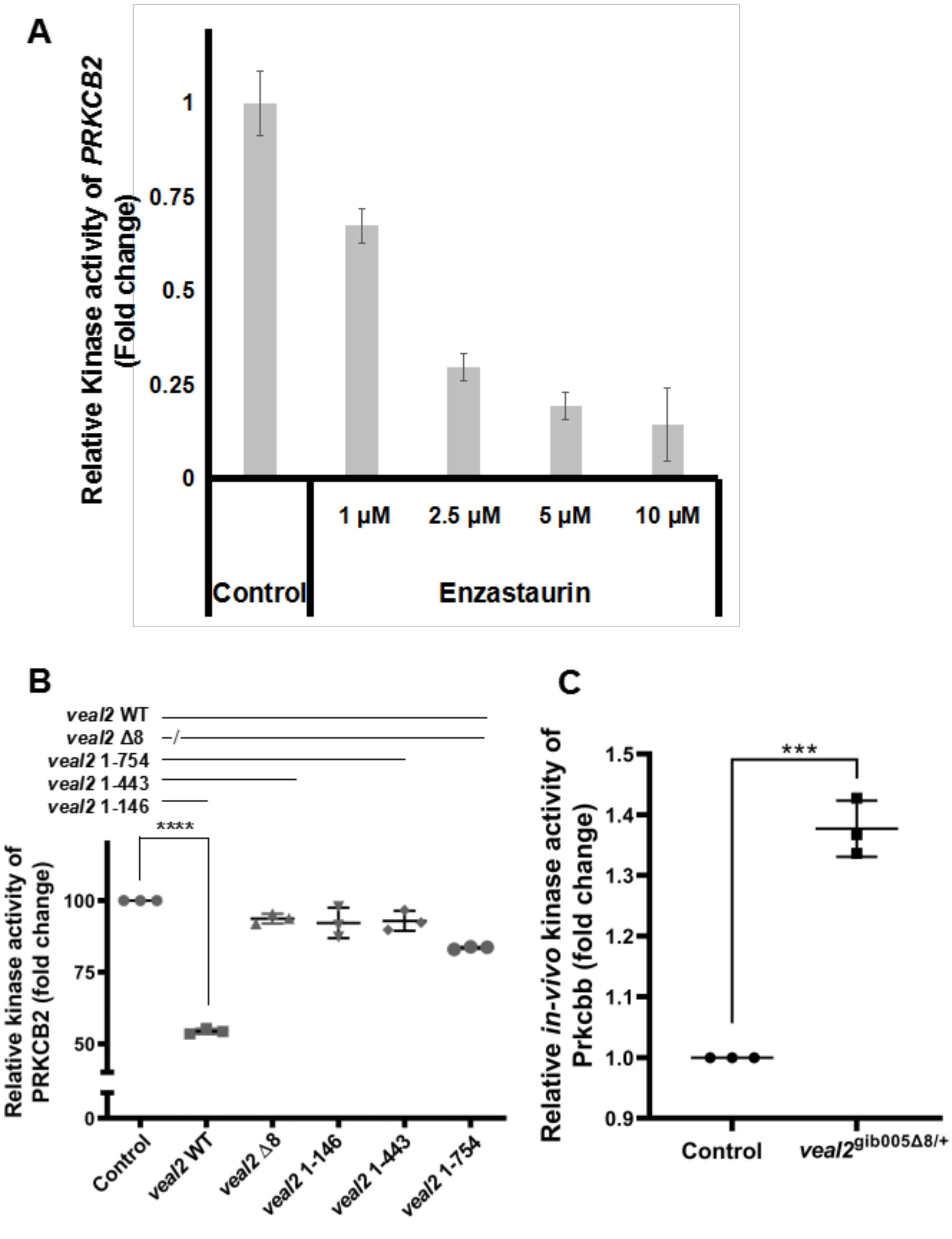

Appendix figure S17: Enzastaurin and full-length *veal2* inhibit the *in vitro* kinase activity of human PRKCB2 protein

A. The bar graph represents the relative change in kinase activity of human PRKCB2 protein upon Enzastaurin treatment. The inhibition of kinase activity is dependent on the dose of Enzastaurin treatment. Data was collected from 3 independent experiments and represented as mean percentage ± standard deviation.

B. Relative kinase activity of human PRKCB2 under standard conditions and in the presence of various fragments of the WT *veal2* IVT RNA. Data from three different experiments plotted as mean fold change values ± standard deviation.

C. Dot plot represents *in-vivo* endogenous kinase activity of Prkcbb in control and in *veal2*$^{gib005\Delta8/+}$ animals. Data from three different experiments plotted as mean fold change values ± standard deviation.

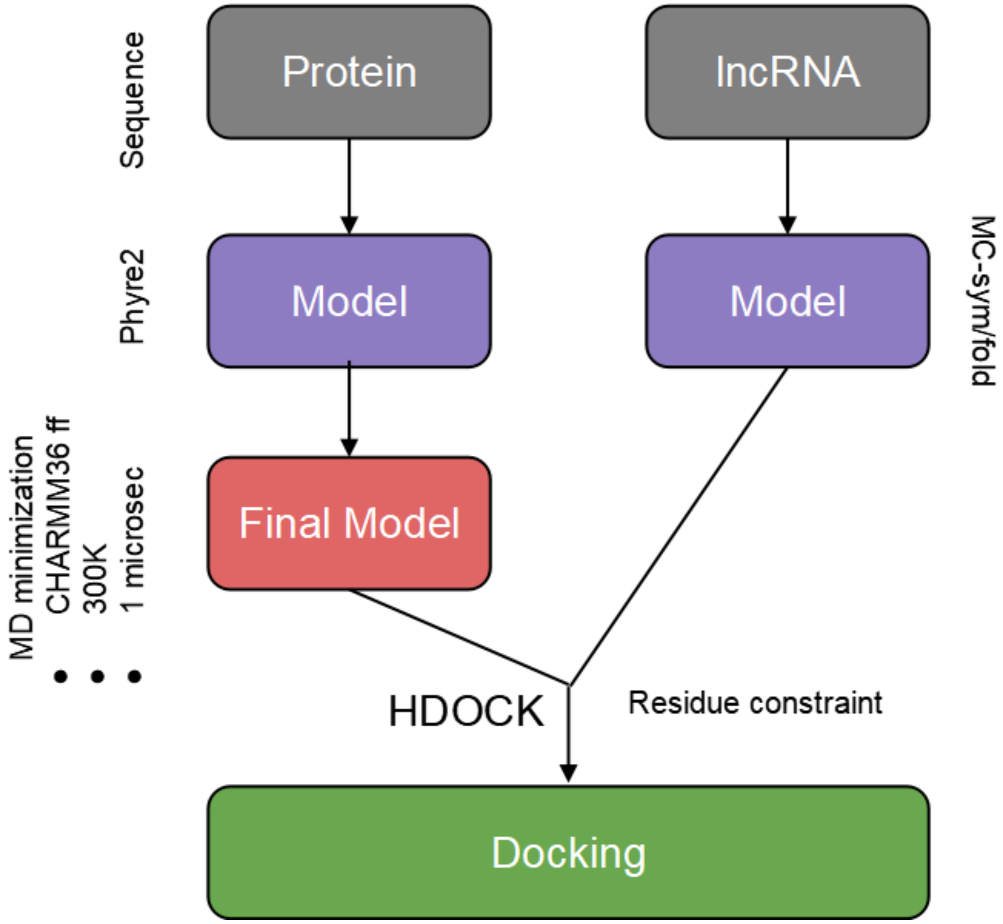

Appendix figure S18: Workflow utilized for docking of *veal2* and zebrafish Prkcbb protein.

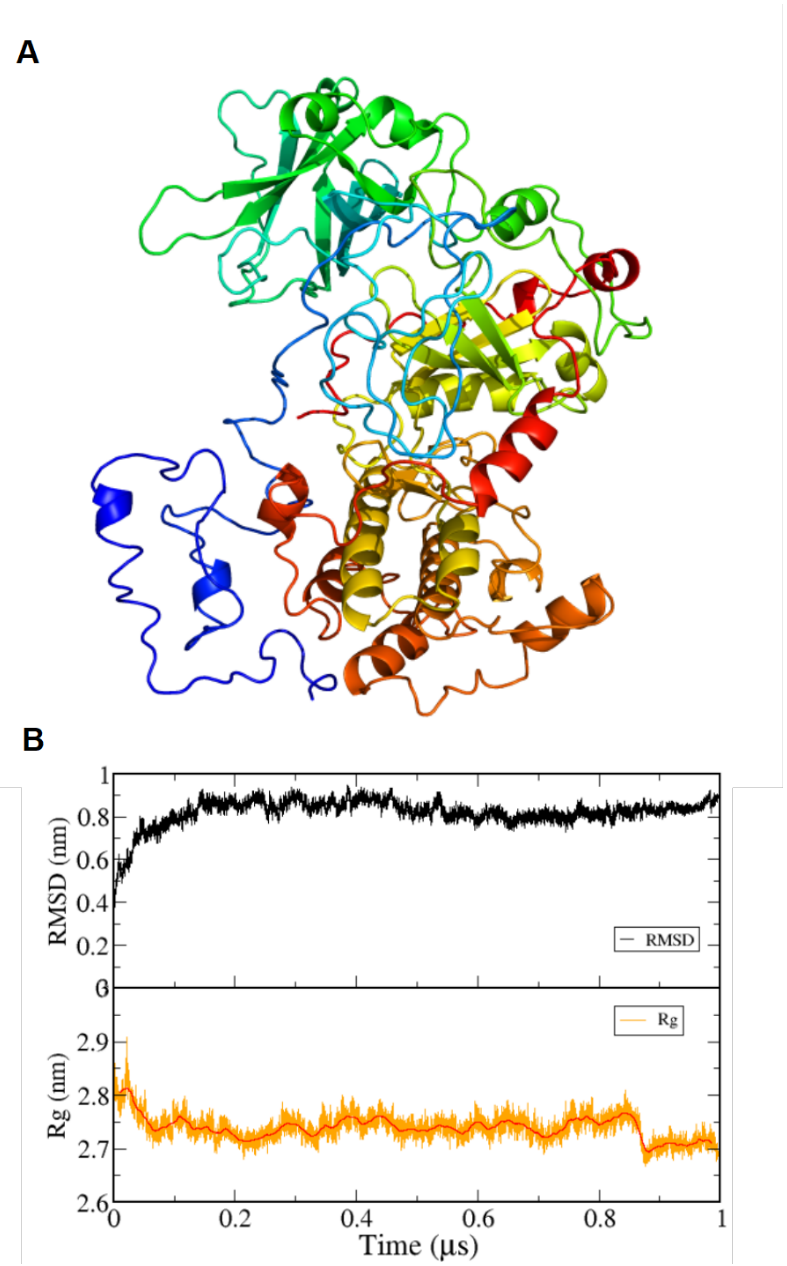

Appendix figure S19: A. Elucidating the structure of kinase protein Prkcbb. The initial modeled structure was obtained using Phyre2.

B. All atomistic molecular dynamics simulation was performed to have a final refined structure. Two order parameters, namely, root mean square deviation (RMSD) and radius of gyration (Rg), showed a stable conformation of the protein after 1 microsecond.

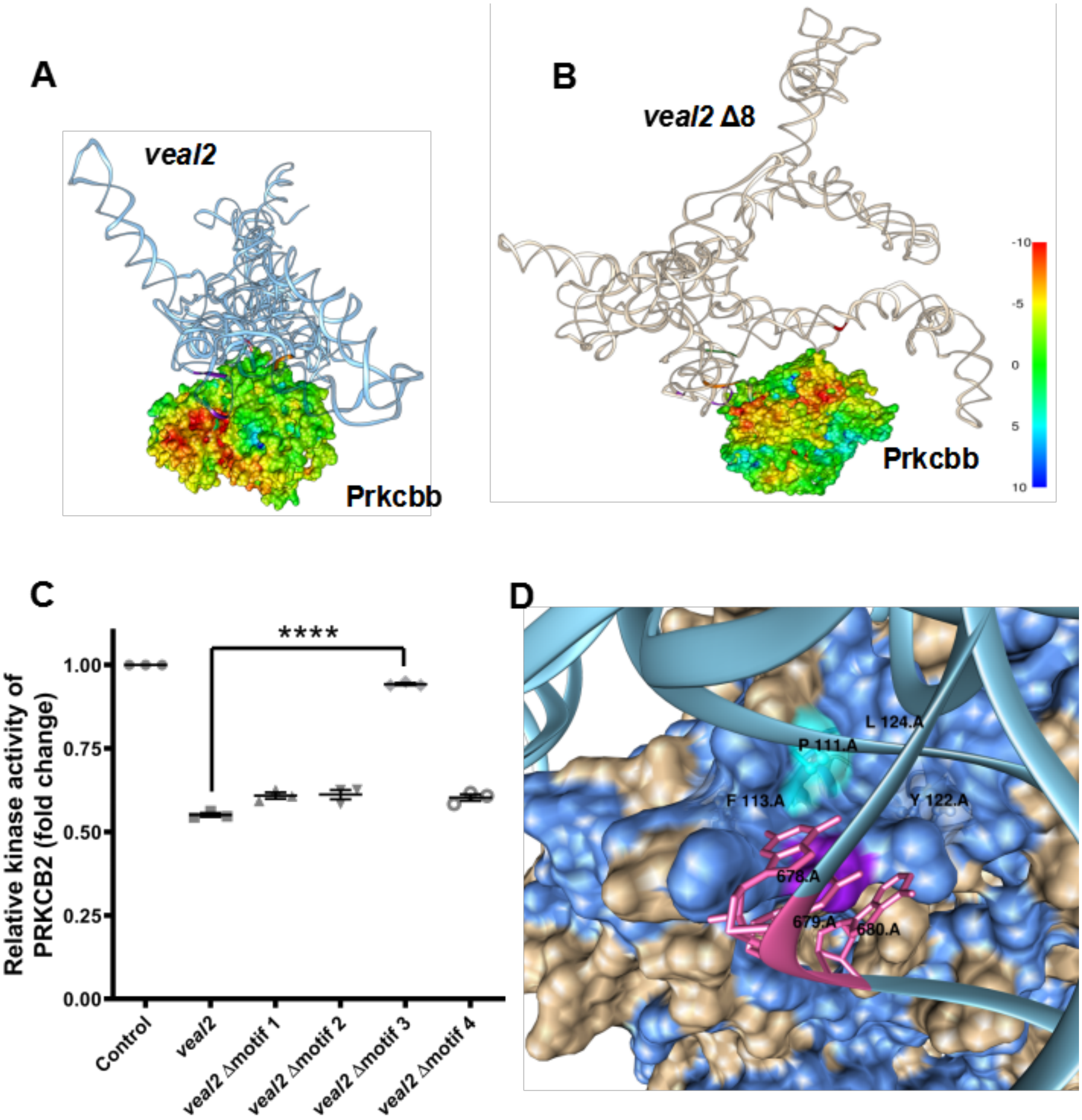

Supplementary Figure 20. Representation of the site of interaction of *veal2* with the Prkcbb protein.

A-B. Representation of *veal2* WT and mutant *veal2*-Δ8 RNA with Prkcbb protein. Four motifs were predicted in *veal2* interacting with Prkcbb, highlighted in different colours. Color scale key represents hydrophobicity in the protein structure.

C. *In-vitro* **r**elative kinase activity of human PRKCB2 under standard conditions and in the presence of various variants of *veal2* lacking putative functional motifs and wt *veal2* IVT RNA. Data from three different experiments plotted as mean fold change values ± standard deviation.

D. The 3 bases of motif 3 (CLM) in *veal2* are highlighted in pink and the base positions are mentioned. The 4 amino acids known to bind with DAG[6] within C1 domain of Prkcbb have been highlighted.

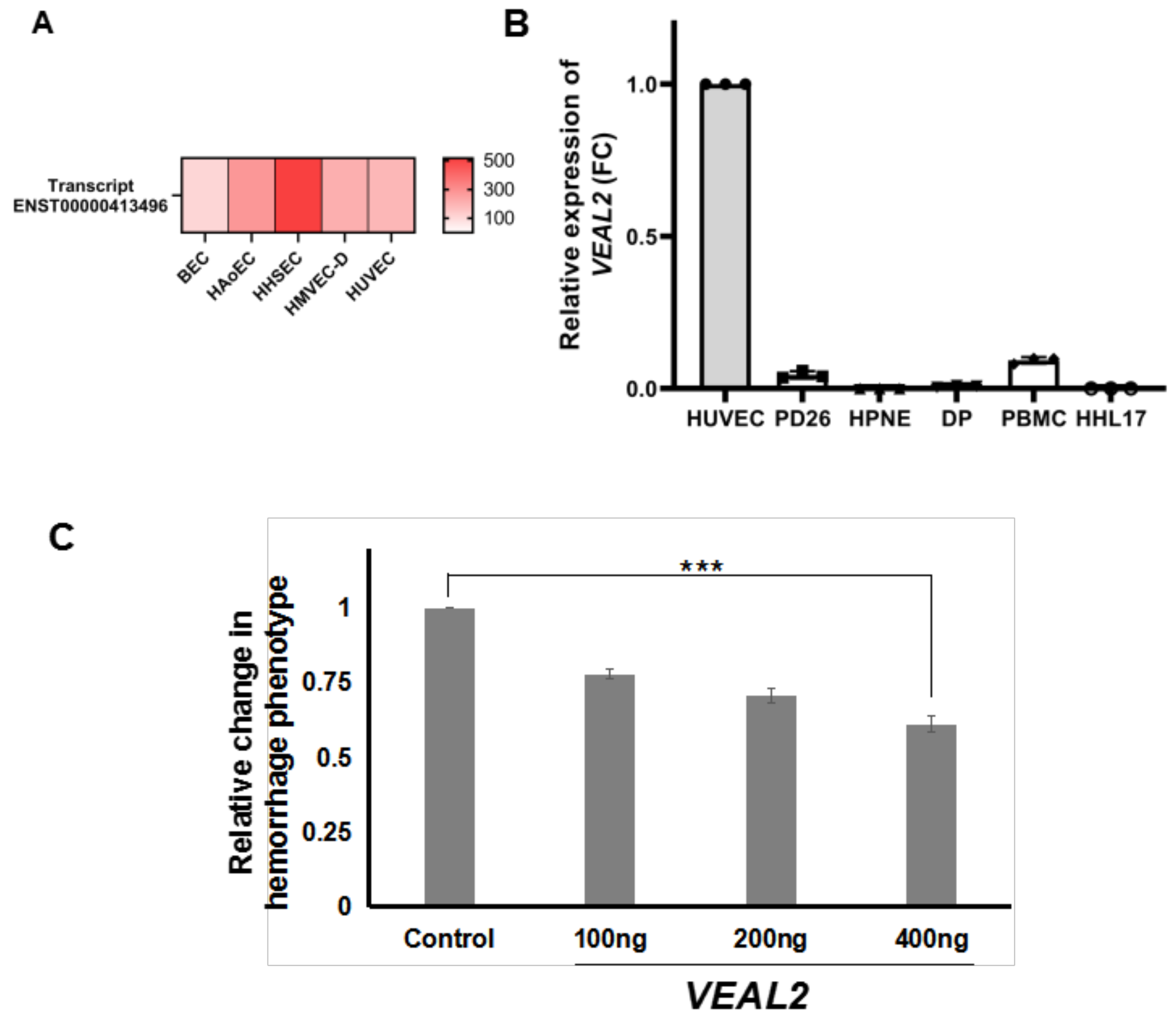

Appendix figure S21. Ortholog of *veal2* in humans can rescue *veal2*$^{gib005\Delta8/+}$ mutant animals.

A. Expression of *VEAL2* in different human primary endothelial cell lines curated by ANGIOGENE (Müller et al, 2016) (http://angiogenes.uni-frankfurt.de/transcript/ENST00000413496) BEC(Brain endothelial cells), HAoEC (Human Aortic Endothelial Cells), HHSEC (Human Hepatic Sinusoidal Endothelial Cells), HMVEC-D (Human Dermal microvascular endothelial cells), HUVEC (Human umbilical vein endothelial cells).

B. Bar graph representing *VEAL2* RNA levels significantly high in endothelial cells (HUVECs) compared to other primary cells like Human Foreskin Fibroblast (HFF), Human Pancreatic Nestin Expressing cells (HPNE), Human Epidermal Melanocytes Neonatal Darkly Pigmented (HEMn-DP), Primary Human Melanocytes, Peripheral Blood Mononuclear Cell (PBMC), Human Hepatocyte Line 17 (HHL17). Data was collected from 3 biological replicates and is presented as as mean percentage values ± standard deviation.

C. Bar graph representing the number of animals that displayed the hemorrhage phenotype across the progeny of outcross of *veal2*$^{gib005\Delta8/+}$ zebrafish upon complementation of human *VEAL2*. Data from three different experiments plotted as mean percentage values ± standard deviation.

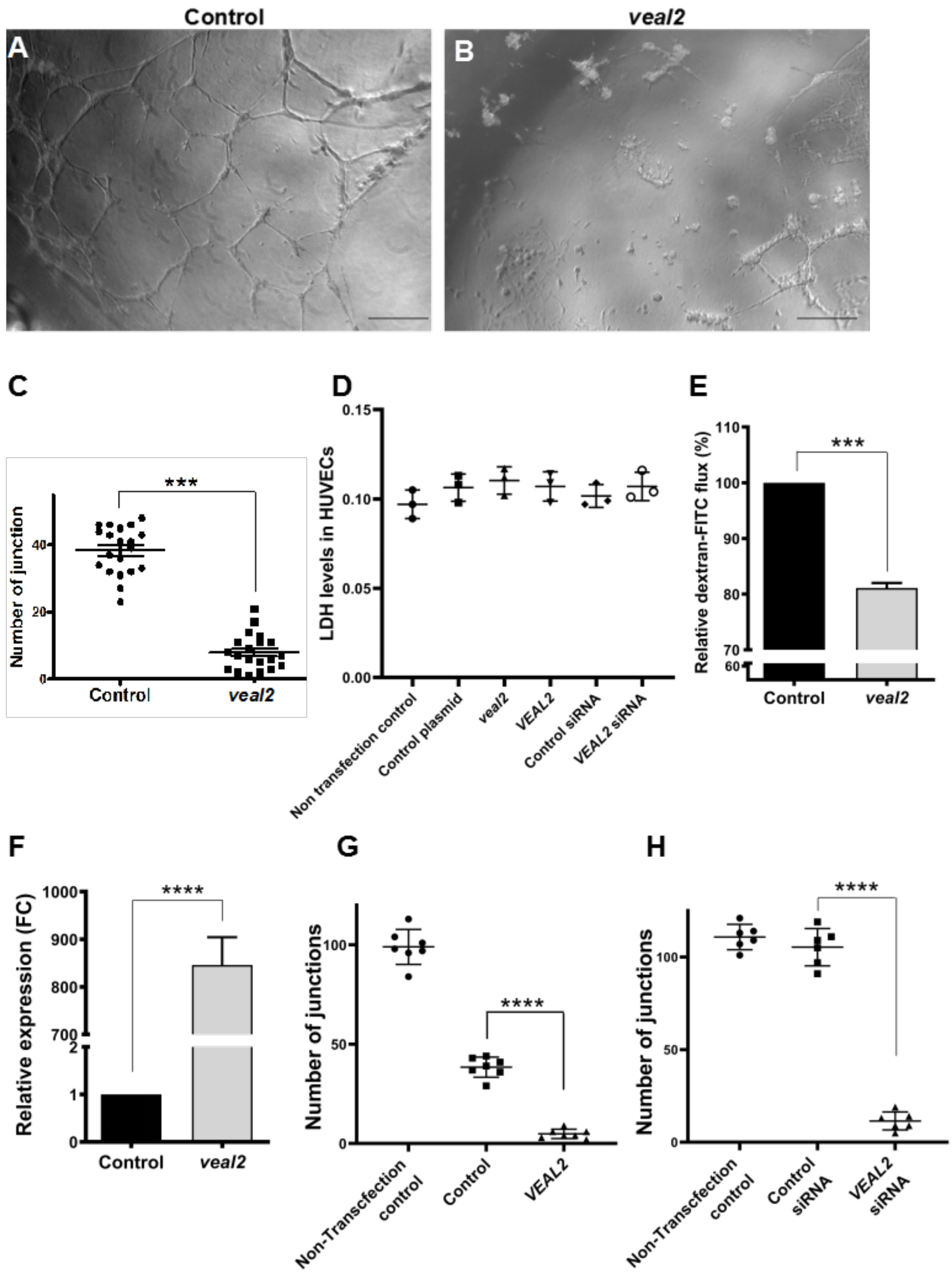
Control
veal2
A
B
C
***
Number of junction
40
20
0
Control
veal2
D
0.15
0.10
0.05
0.00
LDH levels in HUVECs
Non transfection control
Control plasmid
veal2
VEAL2
Control siRNA
VEAL2 siRNA
E
110
100
90
80
70
60
***
Relative dextran-FITC flux (%)
Control
veal2
F
1000
900
800
700
2
1
0
****
Relative expression (FC)
Control
veal2
G
100
50
0
****
Number of junctions
Non-Transcfection control
Control
VEAL2
H
100
50
0
****
Number of junctions
Non-Transcfection control
Control siRNA
VEAL2 siRNA

Appendix figure S22. Overexpression of *veal2* regulates tubulogenesis and permeability in HUVECs.

A-B. Representative images of network of tubes of HUVECs formed in matrigel coated multiwell plate displaying massive reduction in vessel formation upon overexpression of *veal2* in HUVECs. **A.** HUVECs transfected with empty pcDNA3.1. **B.** HUVECs transfected with pcDNA3.1 containing full-length *VEAL2*. **A-B.** 5X magnification with scale bar representing 50μm.

C. Dot plot representing quantification of number of junctions formed between the vessels in control cells and *veal2* overexpressed cells grown in matrigel. Data from different fields of 3 different technical replicates of 3 biological replicates each.

D. Dot plot representing lactate dehydrogenase levels measured in HUVECs transfected with different overexpression plasmids or siRNAs. Data is acquired from 3 different biological replicates and shown as mean fold change values ± standard deviation.

E. Bar graph representing relative quantification of efflux of dextran conjugated FITC measuring permeability levels in control and *veal2* overexpressed HUVEC cells. Data obtained from 2 technical replicates of 2 different biological replicates and plotted as mean fold change values ± standard deviation**.**

F. Bar graph representing relative expression of *veal2* in control and *veal2* overexpressed HUVECs. Data is acquired from 3 different biological replicates and shown as mean fold change values ± standard deviation.

G. Dot plot representing massive reduction in tube formation in matrigel upon overexpression of *VEAL2* in HUVECs compared to control.

H. Dot plot representing massive reduction in tube formation in matrigel upon siRNA mediated knockdown of *VEAL2* in HUVECs compared to control.

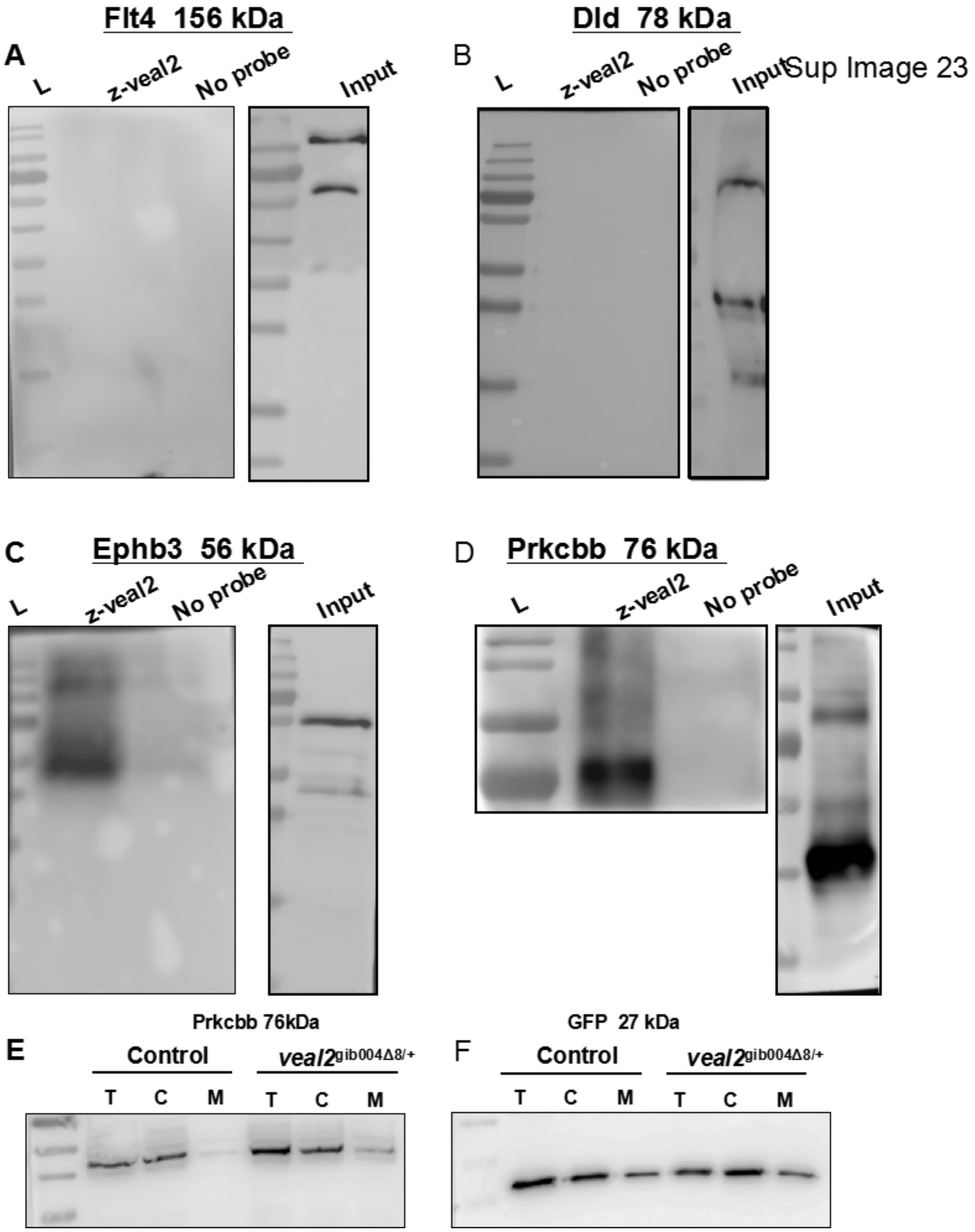

Appendix figure S23. A-D. Full western blot images used in Main Figure 3 for demonstrating protein-*veal2* interaction. In panel D blot was cut before detection using chemiluminescence to increase specificity of the signal. **E-F.** Full western blot images used in Main Figure 4 for demonstrating sub cellular localization of Prkcbb.

Appendix tables

Appendix table S1:Summary of the reads mapped to zebrafish genome

Sample	Total Raw Reads (x2)	Total Filtered Reads (x2)	Percent reads mapped
EC	22,196,648	13,522,122	81.6%
NEC	25,682,141	18,621,685	82.1%

Appendix table S2: List of sequences of morpholino and TALE arms designed to target *veal2*

**ID**	**Sequence 5'-3'**
veal2 Morpholino	GGAGTCTTCATGAACTACAAGTACA
veal2 5’ Left TALE1	CATTAGGAAACCCCA
veal2 5’ Right TALE1	AAACCCGTCAATGTG
veal2 3’ Left TALE2	CCGATTGCACTGCAT
veal2 3’ Right TALE2	GGGAGTTGAGATAGG
VEAL2- siRNA 1	GAAUUGGGAAGGAAAGAAAUU

VEAL2- siRNA 2	GAAAACAGGAUGUGGGCAAUU
VEAL2- siRNA 3	GGAAACAGCAAGAGGAAAGUU
VEAL2- siRNA 4	AAGGAAACAGCAAGAGGAAUU

Appendix table S3: List of primers used

veal2 RT_F	AACCCCATATGAAGCATGCT
veal2 RT_R	AACAGCTTCCAGGTCACACA
veal2 ISH_F	GGCTCATTAGGAAACCCCAT
veal2 ISH_R	TTAAGGAAAGTTGCACTGCCA
*veal2*_full lenght_F	GGCTCATTAGGAAACCCCAT
*veal2*_full lenght_R	TGAACACATGTGAAATTCAGAG
*veal2*_fragemtents_F	GGCTCATTAGGAAACCCCAT
*veal2*_fragementt1_R	TAGAGTACAAAGATTGACCGG
*veal2*_fragemtent2_R	CTGCGGTCACATTAAAGTCAA
*veal2*_fragementt3_R	TCACTCCATTGAGTGTATCGA
*veal2*_motif1_F	ACCAGCTCAACACCTGCACT
*veal2*_motif1_R	CATTGCAATGGTCTTTCATGAT

*veal2*_motif2_F	ATCTATCCACCTGCTGTCAC
*veal2*_motif2_R	AGTGCAGGTGTTGAGCTGGT
*veal2*_motif3_F	GCTTTTATGATGCAAGCTGAG
*veal2*_motif3_R	AGATACTATTAAAGTTGCCAGG
*veal2*_motif4_F	CAGACCCCTCAACTCTATCCA
*veal2*_motif4_R	ACTCTATGCAGTGCAATCGG
*veal2*_RAP_1	GTGTAACTGCCCAAACCGAGCATGCTTCATATGGGGTTTCC TAATGAGCC
*veal2*_RAP_2	CAGCTCTCGTTGACCTTTACTAAAACATATCTGCTAACTTCC ATTCGTCC
*veal2*_RAP_3	CAGTCATATTTCAGTGTGGAGGAACATCGTCTTTACTCCAAA CAGCTTCC
*veal2*_RAP_4	TATCCCAAGGAGAGCTCAGTCCCTCCAATGTGGACTTTCCT CCTGGTAGG
*veal2*_RAP_5	GTCAATCCATCTTCAACCAGAACGGTGGCCAAATCATGGAG GTCATCTAA
*veal2*_RAP_6	GCAGATCCTCCCTCAAAGTGGAGTCTTCATGAACTGCGGTC ACATTAAAG
*veal2*_RAP_7	GATGGCCTGCAGGTCTTTATCCAGAGGTTAAAGCAGCACAA CCTTTTTGA

*veal2*_RAP_8	AGATTTCCACCAGTGCAGGTGTTGAGCTGGTTCAGTCAGAGGCTCATTGC
*veal2*_RAP_9	AGTTGCCAGGATAAAACCTGTAGTGAACAAAAGGGATCTATAAAACGG
*veal2*_RAP_10	AAAAACAGCAACTTAAGGAAAGTTGCACTGCCAAAACAAAACCCTTGTGG
*veal2*_RAP_11	GGCACCAACATGAACCCATACTCTGAGAACCTCTTGGAGGAATGAACAAA
*veal2*_RAP_12	CTGTGAACACATGTGAAATTCAGAGGTAAATTGTGAATTGCTGGACAG
*veal2*_HMA_F	TCCTTTCAGCTCTCGTTGAC
*veal2*_HMA_R	TAGCAACGAGTGCCATGTGT
*veal2*_OT2_F	AAATCCCTACTGTTGAGCCC
*veal2*_OT2_R	ATTCGACTCTGGCATCCTAA
*veal2*_OT3_F	GGGGTTTCTTAATGGCAAAG
*veal2*_OT3_R	GGCTAATAAGCAGTCTAAGG
*veal2*_OT4_F	GGTGTACATAAGAACTGCTG
*veal2*_OT4_R	ACTCTGTTCGTTCATGCACC
*veal2*_OT5_F	TTGGGAGTAGCATTCTGCCT

*veal2*_OT5_R	CTGTGGTGCTGATCAGCTAT
*veal2*_OT6_F	TTTCAGCTAGGAATGGCCAT
*veal2*_OT6_R	CTACCCATATTTACTCCATG
*veal2*_OT8_F	GTTTGCCATTGTACAGGTGG
*veal2*_OT8_R	CTTGCTATAGAACAGCTGGC
*veal2*_del8_Full Length_F	GGCTCATTAGGAAACCCCATATGAAGGTTTGGGCA
*veal2*_del8_Full Length_R	TGAACACATGTGAAATTCAGAG
*VEAL2*_RT_F	GCCAAAGCAGGACACGTTAT
*VEAL2*_RT_R	TGTTTTCACCCCTGAGCTGT
*VEAL2*_FL_F	GCCAGGAAAAAGAAAGAAAAAC
*VEAL2*_FL_R	CTTTTTCTTTCCTTCCCAATTC
veal2-GSP-1R	CTACCCATATTTACTCCATG
veal2-GSP-2R	CTGTGGTGCTGATCAGCTAT
veal2-GSP-3R	ACTCTGTTCGTTCATGCACC
veal2-GSP-4R	GGCTAATAAGCAGTCTAAGG
veal2-GSP-1F	GGGGTTTCTTAATGGCAAAG
veal2-GSP-2F	GGTGTACATAAGAACTGCTG

VEAL2-GSP-1F	GCCAAAGCAGGACACGTTAT
VEAL2-GSP-2F	ACATCGCTGTCCCTCACAAA
VEAL2-sm1	CCCGCTTTCAAGGCAAGA
VEAL2-sm2	GAATAACGTGTCCTGCTT
VEAL2-sm3	ATGTGGCCCTCTGTGTTA
VEAL2-sm4	GTATTTGTGAGGGACAGC
VEAL2-sm5	GGACGAAGAACCACAGGA
VEAL2-sm6	CACATCCTGTTTTCACCC
VEAL2-sm7	CCTTGCACTCTAACCAGT

Appendix table S4: Expression of neighboring genes in *veal2* loci in *veal2*$^{gib005\Delta8/+}$ zebrafish embryos

			Expression (FPKM)		
**Gene**	**Location w.r.t *VEAL2***	**Locus**	**Control**	***veal2*$^{gib005\Delta8/+}$**	**log2(FC)**
cyb5b	Downstream	chr7:67510406-67521267	13.4503	14.8792	0.145652
dhx38	Downstream	chr7:67525898-67574013	7.93762	7.59586	-0.0634929
zgc:162592	Upstream	chr7:67475825-67483397	0.030225	0.0135615	-1.15626
kars	Upstream	chr7:67206104-67225532	81.5742	83.6073	0.0355163
rpl13	Upstream	chr7:67244349-67253785	1075.5	934.546	-0.202673

Appendix table S5: *veal2* pull-down (PD) specific proteins identified to be enriched in "Angiogenesis" pathway.

	***veal2* PD 1**	***veal2* PD 2**	***veal2* PD 3**	**Control PD 1**	**Control PD 2**	**Control PD 3**	**p-value**	**FC1**	**FC2**	**FC3**
Dllc_DANRE	42	60	56	0	13	0	0.007	43	4.35	57
Wnt1_DANRE	26	37	37	0	23	0	0.060	27	1.58	38
Dlld_DANRE	23	40	39	0	12	0	0.023	24	3.15	40
Axin2_DANRE	20	33	50	0	27	0	0.190	21	1.21	51
Ephb3_DANRE	12	23	28	0	17	0	0.144	13	1.33	29
Efnb2_DANRE	9	22	20	0	5	0	0.042	10	3.83	21
Vgfr4_DANRE	39	62	71	4	17	0	0.042	8	3.5	72

Tie2_DANRE	29	39	48	3	12	0	0.042	7.5	3.07	49
**Kpcb/ Prkcbb_DANRE**	**38**	**47**	**42**	**2**	**0**	**3**	**0.006**	**13**	**48**	**10.7 5**
Vgfr3_DANRE	32	73	72	25	53	0	0.238	1.26	1.37	73

Appendix table S6: Differential expression of protein coding genes across EC and NEC RNAseq datasets.

**Gene Sym**	**Gene Name**	**GFP+ FPKM**	**GFP- FPKM**
**Upregulated genes**			
flt4	fms-related tyrosine kinase 4	22.6425	1.32247
tbx6	T-box gene 6	34.6608	5.16525
kdrl	kinase insert domain receptor like	14.9846	1.59662
dlx2a	distal-less homeobox gene 2a	11.9818	6.99252
kdr/flk1	kinase insert domain receptor	4.29871	1.72571E-05
fli1a	Friend leukemia integration 1 transcription factor	29.1922	0.342128
**Down regulated genes**			

ptprn2	protein tyrosine phosphatase, receptor type, N polypeptide 2	1.02787	17.2154
dlb	deltaB (dlb)	9.72498	77.2004
**Other examples**			
npsn	nephrosin	182.974	4.92554
hhex	Hematopoietically expressed homeobox (hhex)	99.8801	3.09114
mir130c-1	Mir130c-1	8.19273	0
tie1	Endothelium-specific receptor tyrosine kinase 1	7.81131	0.179456
vap	Vascular associated protein	3.90665	0

References

1. Aday AW, Zhu LJ, Lakshmanan A, Wang J, Lawson ND. Identification of cis regulatory features in the embryonic zebrafish genome through large-scale profiling of H3K4me1 and H3K4me3 binding sites. Dev Biol. 2011;357(2):450-62.
2. Bogdanovic O, Fernandez-Miñán A, Tena JJ, de la Calle-Mustienes E, Hidalgo C, van Kruysbergen I, van Heeringen SJ, Veenstra GJ, Gómez-Skarmeta JL. Dynamics of enhancer chromatin signatures mark the transition from pluripotency to cell specification during embryogenesis. Genome Res. 2012;22(10):2043-53.
3. Collins JE, White S, Searle SM, Stemple DL. Incorporating RNA-seq data into the zebrafish Ensembl genebuild. Genome Res. 2012 Oct;22(10):2067-78.
4. Irimia M, Tena JJ, Alexis MS, Fernandez-Miñan A, Maeso I, Bogdanovic O, de la Calle-Mustienes E, Roy SW, Gómez-Skarmeta JL, Fraser HB. Extensive conservation of ancient microsynteny across metazoans due to cis-regulatory constraints. Genome Res. 2012;22(12):2356-67.
5. Leonard TA, Różycki B, Saidi LF, Hummer G, Hurley JH. Crystal structure and allosteric activation of protein kinase C βII. Cell. 2011;144(1):55-66.
6. Müller R, Weirick T, John D, et al. ANGIOGENES: knowledge database for protein-coding and noncoding RNA genes in endothelial cells. *Sci Rep*. 2016;6:32475.
7. Muruganujan A, Huang X, Ebert D, Mills C, Guo X, Thomas PD. Protocol Update for large-scale genome and gene function analysis with the PANTHER classification system (v.14.0). Nat Protoc. 2019 Mar;14(3):703-721. Doi: 10.1038/s41596-019-0128-8. Epub 2019 Feb 25. PubMed PMID: 30804569; PubMed Central PMCID: PMC6519457
