# Supplementary material for: LncRNA VEAL2 regulates PRKCB2 to modulate endothelial permeability in diabetic retinopathy
Source: EMBO J. 2021 Jun 28;40(15):e107134. doi: 10.15252/embj.2020107134 (PMC8327952; doi:10.15252/embj.2020107134)
Supplement: Supplementary file 3 — Expanded View Figures PDF [file EMBJ-40-e107134-s003.pdf]

Expanded View Figures

Figure EV1. Knockdown of *veal2* transcript in zebrafish embryos leads to vessel patterning and integrity defects.

A Schema representing the design of splice-block morpholino on *veal2* transcript and injection into one-cell staged double transgenic *gib004Tg(fli1a:EGFP;gata1a:DsRed)* zebrafish embryos by microinjection (3 nl at 500 μM). The injected embryos were further screened at 2 dpf for phenotypic changes.

B–G Representative images of morpholino-injected zebrafish at 2 dpf under bright field and EGFP filter. (B, D, F) Embryos injected with scrambled morpholinos. (C, E, G) Embryos injected with the *veal2* morpholino. *veal2* knockdown induces sprouting defects (indicated by arrowheads). (B–E) 5× magnification. Scale bars represent 100 μm. (F–G) 20× magnification. Scale bars represent 50 μm.

H Bar graph representing a number of animals displaying vascular sprouting defects in *veal2* morpholino-injected zebrafish at 2 dpf. Data from three different experiments plotted as mean percentage values ± standard deviation.

I–N Representative images of morpholino-injected 2 dpf zebrafish under bright field, mRFP filter and animals stained with O-dianisidine. (I, K, M) Embryos injected with non-targeting control (NTC) morpholino. (J, L, N) Embryos injected with the *veal2* morpholino. Arrowheads show the presence of hemorrhage due to the vascular integrity defects. (I–N) 5× magnification. Scale bars represent 100 μm.

O Percentage of animals that showed vascular integrity defects at 2 dpf when injected with 3 nl of 500 μM scrambled morpholino, 500 μM *veal2* morpholino and cocktail of 500 μM *veal2* morpholino and 100 ng of *veal2* RNA. Data from three different biological replicates represented as mean percentage ± standard deviation.

P Gel represents the PCR-amplified products using primers designed across the intron. The arrowhead indicates the product with retention of the intron due to the effect of morpholino.

Q Relative expression of *veal2* across control and *veal2* knockdown embryos. *actb* was taken as normalization control. Data from three different experiments represented as mean $\Delta\Delta C_T$ values normalized to EC values ± standard deviation.

Data information: All the experiments $N \geq 3$. ****P*-value < 1E-3 and *****P*-value < 1E-4. Statistics: unpaired two-tailed t-test.

© 2021 The Authors

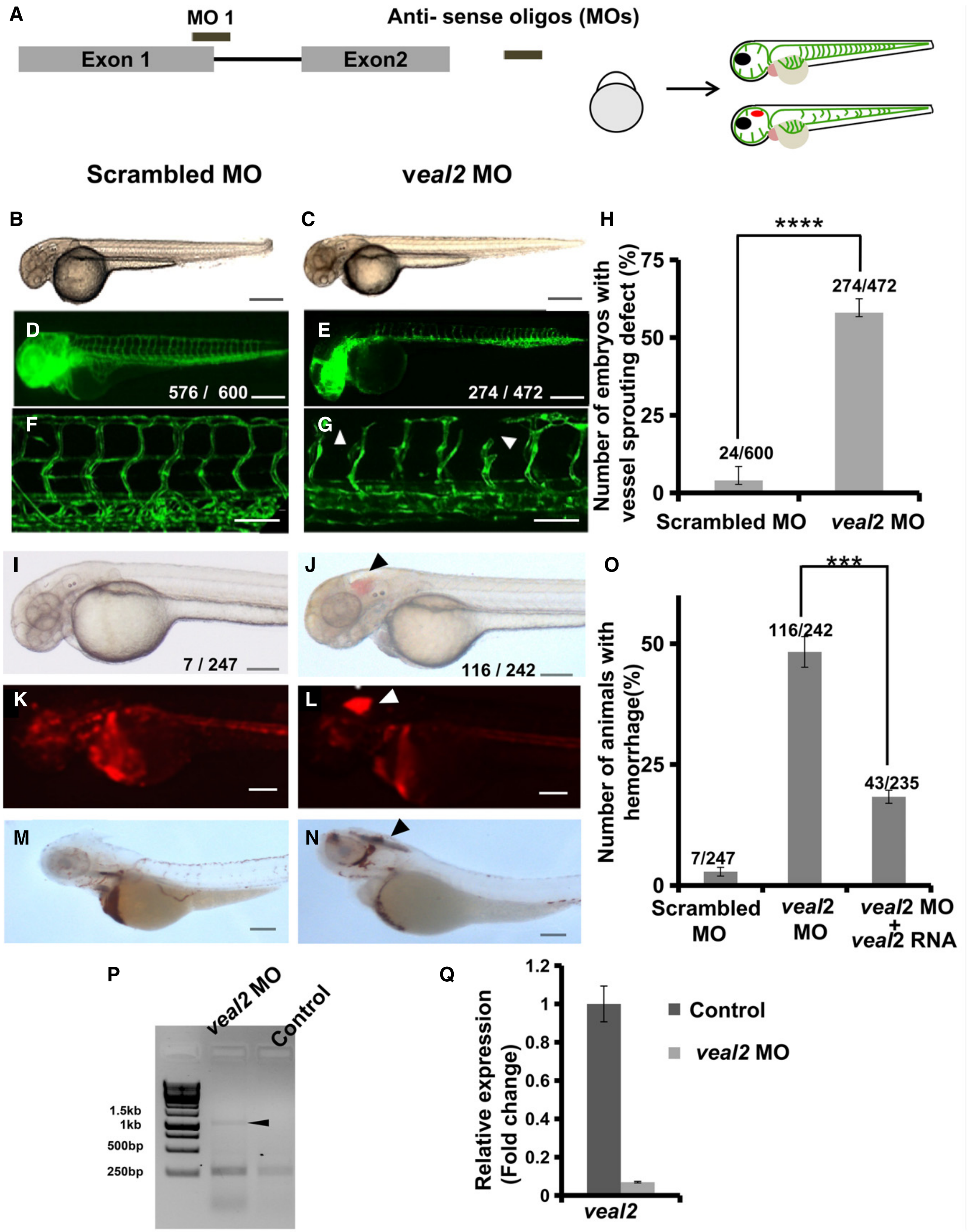
A
MO 1
Anti- sense oligos (MOs)
Exon 1
Exon2
Scrambled MO
veal2 MO
B
C
D
E
576 / 600
274 / 472
F
G
H
****
Number of embryos with vessel sprouting defect (%)
75
50
25
0
274/472
24/600
Scrambled MO
veal2 MO
I
J
7 / 247
116 / 242
K
L
M
N
O
***
Number of animals with hemorrhage(%)
50
25
0
116/242
43/235
7/247
Scrambled MO
veal2 MO
veal2 MO + veal2 RNA
P
veal2 MO
Control
1.5kb
1kb
500bp
250bp
Q
Relative expression (Fold change)
1.2
1
0.8
0.6
0.4
0.2
0
Control
veal2 MO
veal2

Figure EV1.

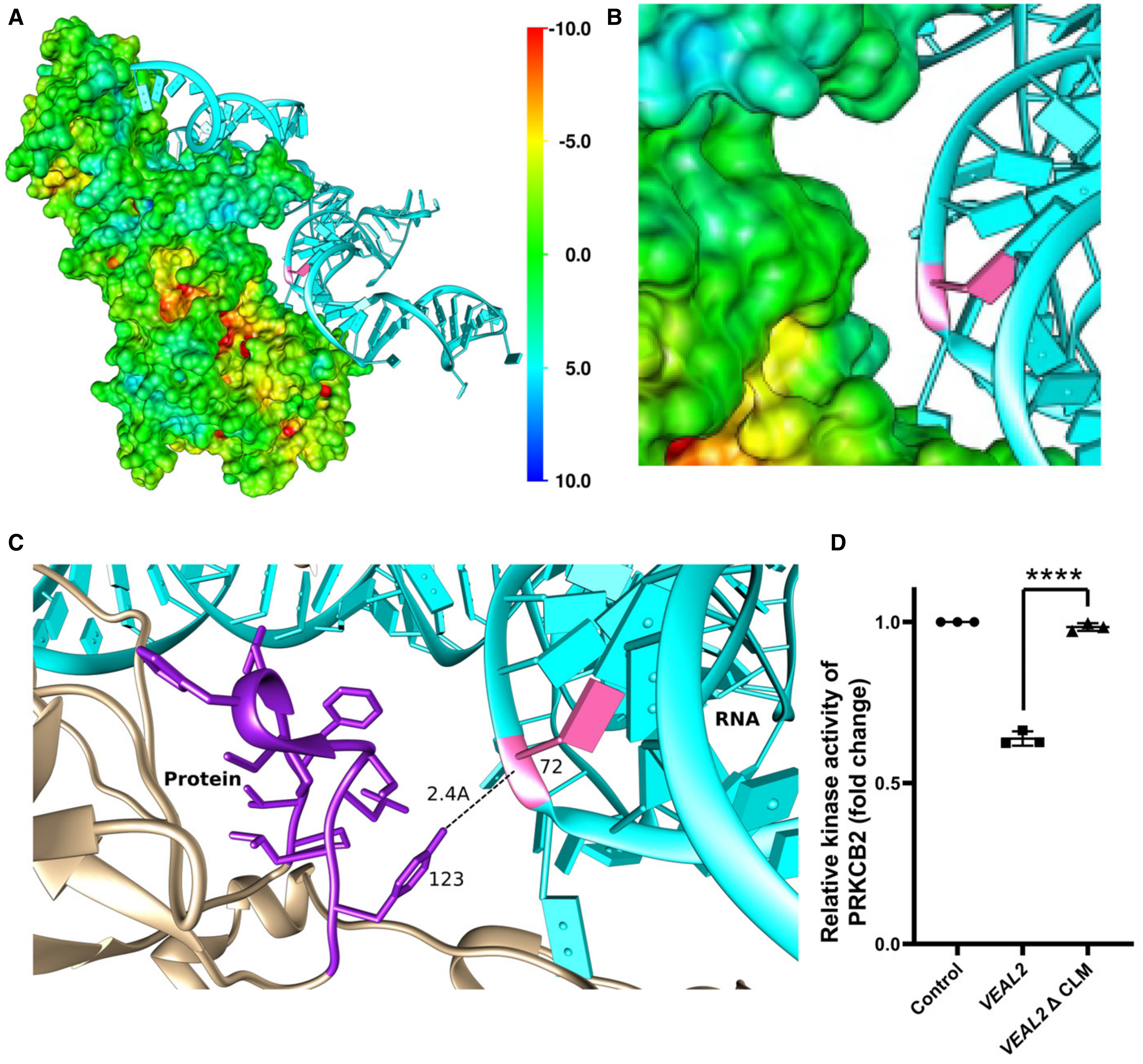

Figure EV2. Representation of the site of interaction of *veal2* with the Prkcbb protein.

A, B Representation of interaction of *veal2* WT RNA with Prkcbb protein. (B) Enlarged view of interaction of motif-3 of *veal2* and Prkcbb protein.

C The 3 bases of motif-3 (CLM) in *veal2* are highlighted in pink, and the base positions are mentioned. The 4 amino acids known to bind with DAG (Leonard *et al*, 2011) within C1 domain of Prkcbb have been highlighted.

D Relative kinase activity of human PRKCB2 under standard conditions and in the presence of various variants of *veal2* lacking putative functional motifs and wt *veal2* IVT RNA. Data from three different experiments plotted as individual values; the middle bar represents the mean, and the error bar represents ± standard deviation.

Data information: All the experiments $N \geq 3$. ****P-value < 1E-4. Statistics: one-way ANOVA with Bonferroni's multiple data comparison

 © 2021 The Authors

Figure EV3. Overexpression and knockdown of *VEAL2* regulate migration and proliferation in HUVECs.

A Representative images showing wound closure rate in overexpressed *VEAL2* and control plasmid-transfected HUVEC monolayer at 0, 9, and 24 h post-scratch. Images taken at 10× magnification with scale bar representing 50 µm.

B Dot plot representing wound closure rate at 0, 9, and 24 h post-scratch in control cells and *veal2*-overexpressed HUVEC monolayer. Data from 3 different technical replicates of 3 biological replicates are presented. Data are plotted as individual values; the middle bar represents the mean, and the error bar represents ± standard deviation.

C Representative images showing wound closure rate in control siRNA- and *VEAL2* siRNA-transfected HUVEC monolayer at 0, 9, and 24 h post-scratch. Images taken at 10× magnification with scale bar representing 50 µm.

D Dot plot representing wound closure rate at initial time, 9, and 24 h post-scratch in control siRNA- and *VEAL2* siRNA-transfected HUVEC monolayer. Data from 3 different technical replicates of 3 biological replicates are presented. Data are plotted as individual values; the middle bar represents the mean, and the error bar represents ± standard deviation.

E, F smFISH of *VEAL2* in HUVECs transfected with control siRNA and *VEAL2* siRNA shows specificity of cytoplasmic signal of *VEAL2*. *VEAL2* in CAL Fluor Red (610 nM). Merged image for *VEAL2* and DAPI. Magnification-100× and scale bar-5 µm.

Data information: All the experiments $N \geq 3$. **P-value < 1E-2 and ***P-value < 1E-3. Statistics: one-way ANOVA with Bonferroni's multiple data comparison.

© 2021 The Authors

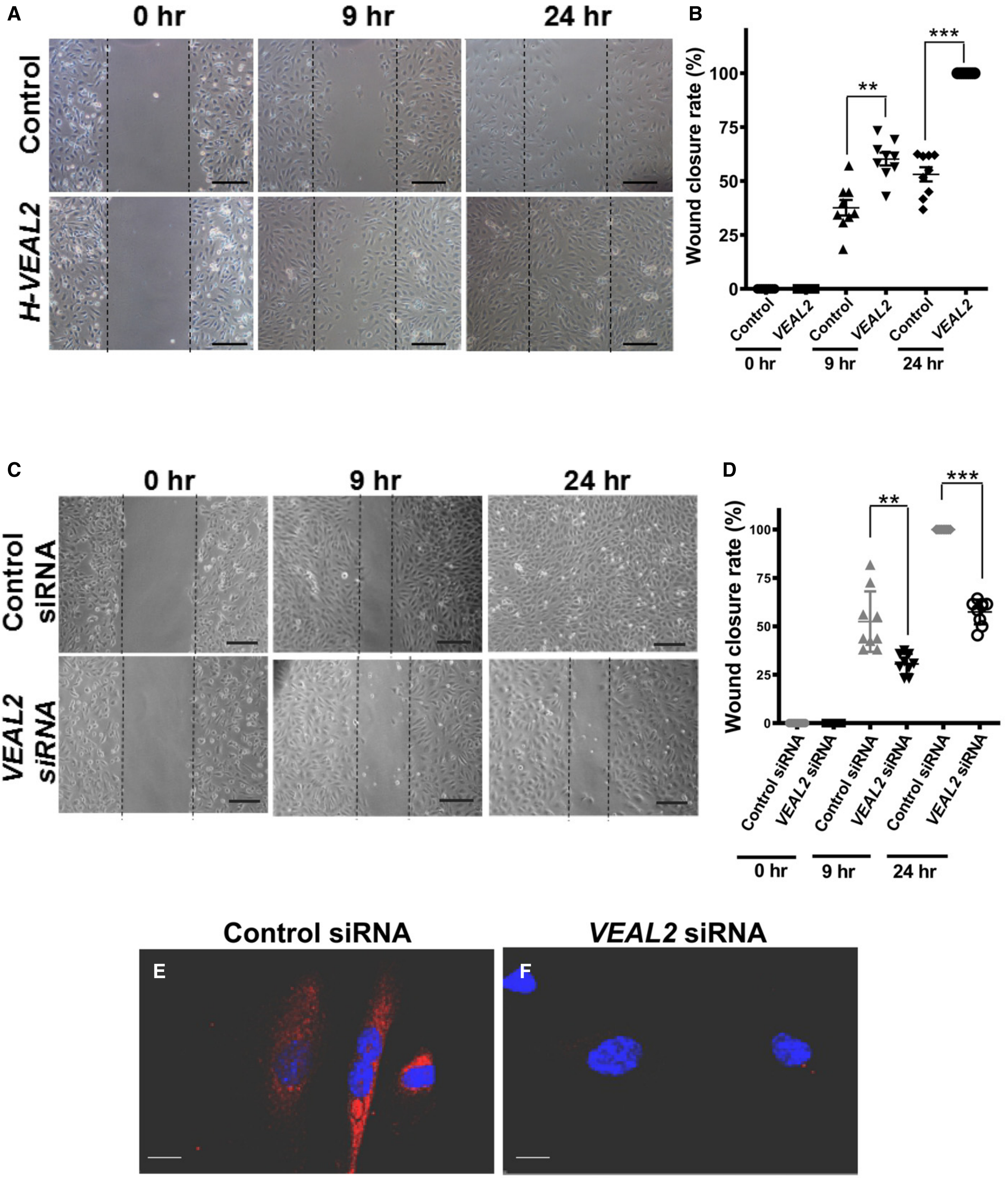

Figure EV3.

 © 2021 The Authors

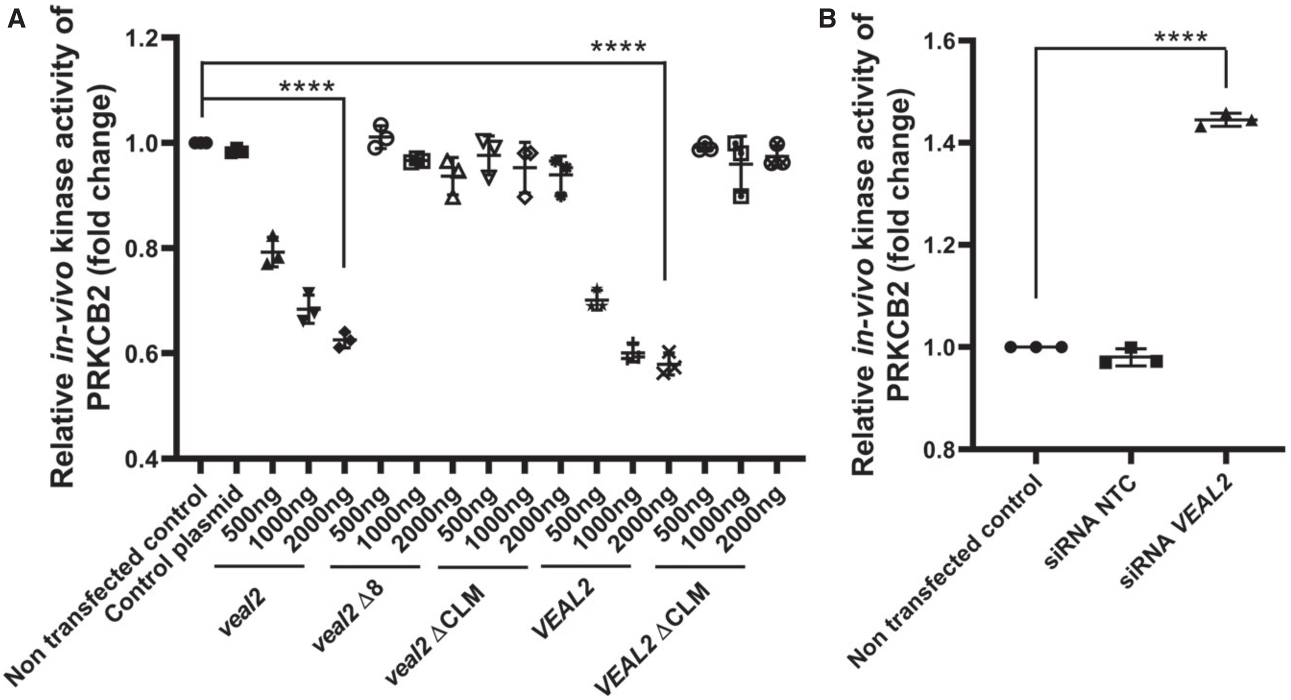

Figure EV4. Identification of regulatory role of *veal2* and *VEAL2* on endogenous kinase activity of PRKCB2 HUVECs.

A Dot plot representing endogenous kinase activity of human PRKCB2 in HUVECs under standard conditions and upon overexpression of various variants of *veal2*, wt *veal2*, *VEAL2*, and its variant IVT RNA. Data from three different experiments are plotted as individual values; the middle bar represents the mean, and the error bar represents ± standard deviation.

B Dot plot representing endogenous kinase activity of human PRKCB2 in HUVECs under standard conditions and upon knockdown of *VEAL2*. Data from three different experiments are plotted as individual values; the middle bar represents the mean, and the error bar represents ± standard deviation.

Data information: All the experiments $N \geq 3$. ****P-value < 1E-4. Statistics: one-way ANOVA with Bonferroni's multiple data comparison.

Figure EV5. Validation of pathophysiology associated to diabetic retinopathy of patient samples.

A, B H&E-based immunohistochemistry of retina indicating symptoms of diabetic retinopathy (DR). (A) Retina of control sample indicating regular retinal structures with proper cellular organization. (B) Retina samples of DM patients highlighted early symptoms of retinopathy in form of degeneration of ganglion layer, microaneurysm, arteriolar dilatation, and mild edema. Scale bar is 200 μm.

C–F Retina scan of patients highlighting symptoms of retinopathy. (C) Fundus fluorescence angiography of patients showing vessel integrity defects. (D) Fundus photograph of a patient with symptoms of proliferative diabetic retinopathy and having subhyaloid hemorrhages. (E) Fundus photograph of a patient with symptoms of proliferative diabetic retinopathy with fibrovascular proliferation at disk and abnormal new blood vessels (NVE). (F) Optical coherence tomography of patients with diabetic macular edema.

G Dot plot representing relative expression of *VEAL2* (in fold change) in fibrous membrane isolated from control and PDR patients. Data obtained from 7 patients as biological replicates and represented as individual values with mean fold change values ± standard deviation.

H Bar graph representing relative expression of *VEAL2* in control of HUVECs and hyperglycemia stimulated HUVECs by growing under high glucose. Data are acquired from 3 different biological replicates and shown as individual values with mean fold change values ± standard deviation.

Data information: All the experiments $N \geq 3$. ****P-value < 1E-4. Statistics: unpaired two-tailed t-test.

▶

© 2021 The Authors

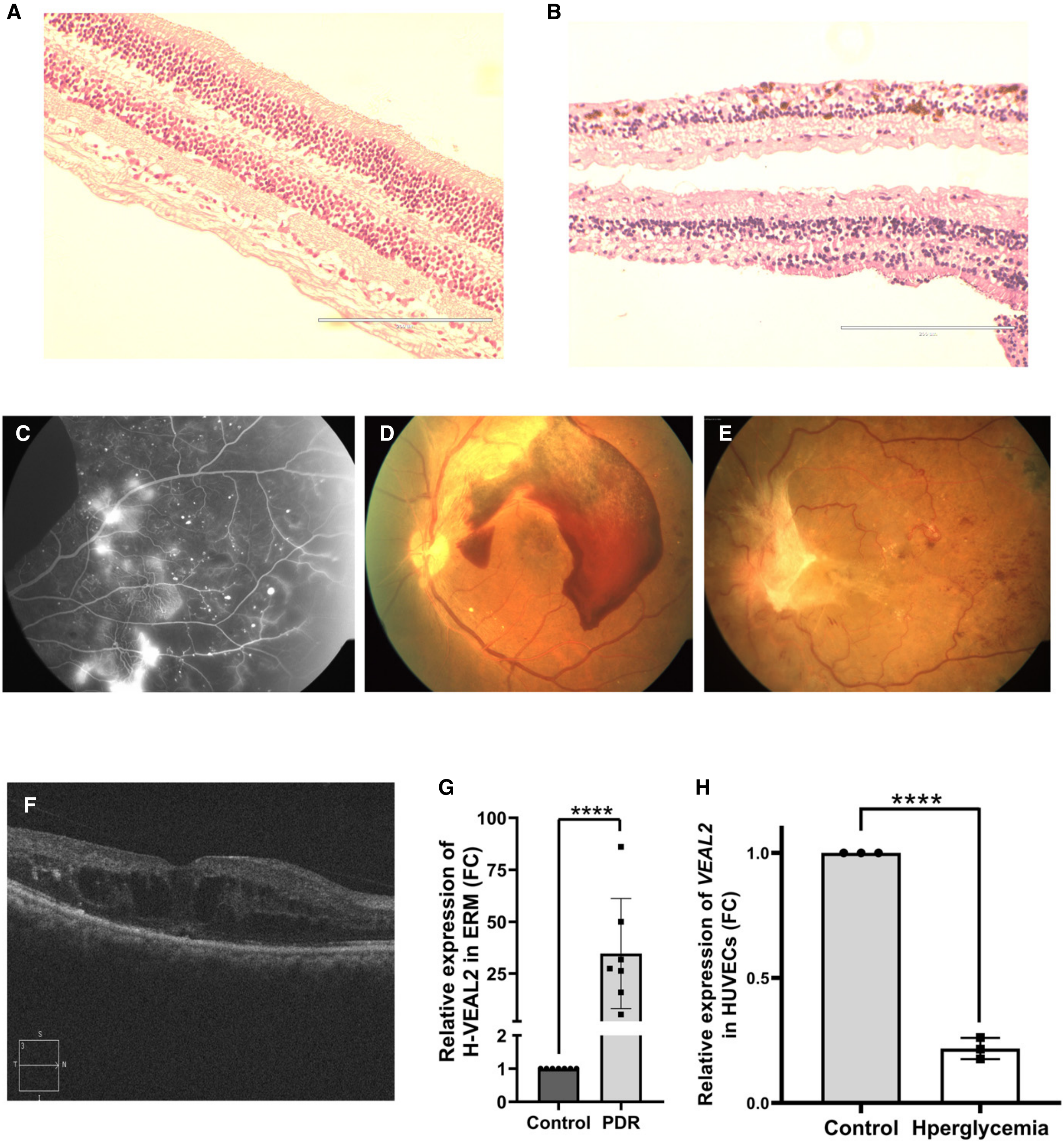

Figure EV5.

 © 2021 The Authors
